# Porphyrinoid rotaxanes: building a mechanical picket fence†
†Electronic supplementary information (ESI) available: Full synthetic procedures and characterisation of all novel compounds. See DOI: 10.1039/c7sc03165c


**DOI:** 10.1039/c7sc03165c

**Published:** 2017-08-04

**Authors:** T. H. Ngo, J. Labuta, G. N. Lim, W. A. Webre, F. D'Souza, P. A. Karr, J. E. M. Lewis, J. P. Hill, K. Ariga, S. M. Goldup

**Affiliations:** a International Center for Young Scientists (ICYS) , WPI Center for Materials Nanoarchitectonics (WPI-MANA) , National Institute for Materials Science , Namiki 1-1 , Tsukuba , Ibaraki 305-0044 , Japan . Email: NGO.Huynhthien@nims.go.jp; b WPI Center for Materials Nanoarchitectonics , National Institute for Materials Science , Namiki 1-1 , Tsukuba , Ibaraki 305-0044 , Japan; c International Center for Young Scientists (ICYS-SENGEN) , National Institute for Materials Science , Sengen 1-2-1 , Tsukuba , Ibaraki 305-0047 , Japan; d Department of Chemistry , University of North Texas , 1155 Union Circle , 305070 , Denton , TX 76203 , USA . Email: Francis.Dsouza@unt.edu; e Department of Physical Sciences and Mathematics , Wayne State College , 111 Main Street , Wayne , Nebraska 68787 , USA; f Department of Chemistry , University of Southampton , University Road , Highfield , Southampton , SO17 1BJ , UK . Email: s.goldup@soton.ac.uk

## Abstract

Building on recent progress in the synthesis of functional porphyrins for a range of applications using the Cu-mediated azide–alkyne cycloaddition (CuAAC) reaction, we describe the active template CuAAC synthesis of interlocked triazole functionalised porphyrinoids in excellent yield. By synthesising interlocked analogues of previously studied porphyrin–corrole conjugates, we demonstrate that this approach gives access to rotaxanes in which the detailed electronic properties of the axle component are unchanged but whose steric properties are transformed by the mechanical “picket fence” provided by the threaded rings. Our results suggest that interlocked functionalised porphyrins, readily available using the AT-CuAAC approach, are sterically hindered scaffolds for the development of new catalysts and materials.

## Abstract 2

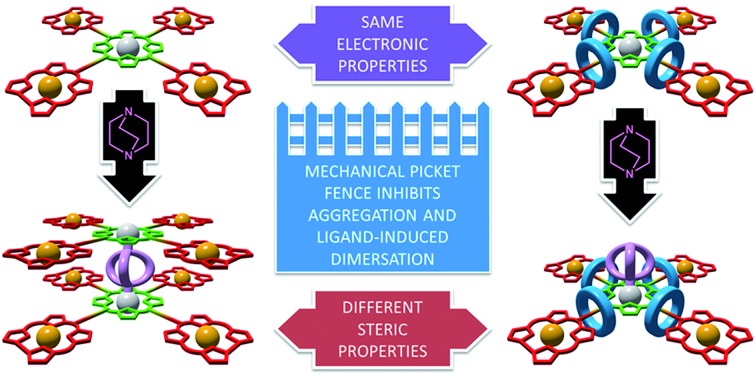
We demonstrate that the threaded macrocycles in interlocked porphyrin–corrole conjugates provide a mechanical “picket fence” without affecting their electronic properties.

## Introduction

The copper-mediated alkyne–azide cycloaddition (CuAAC)^1^ reaction has been widely applied in the synthesis of functionalised porphyrinoid macrocycles,^2^ including multi-porphyrinoid arrays,^3^ for applications in biology,^4^ energy transfer,^5^ catalysis,^6^ self-assembly,^7^ and sensing.^8^ Indeed, the broad functional group tolerance, mild conditions and readily available starting materials of this archetypal “click”^9^ reaction make it an ideal tool for the synthesis of complex non-natural products.

The CuAAC reaction has also been widely applied in the synthesis of interlocked molecules,^10^ including examples of rotaxanes and catenanes containing porphyrin sub-units.^11^,^12^ To achieve this, triazole formation is often the final step that captures the interlocked structure by introducing a stopper unit in the case of rotaxanes or closing a macrocycle in the case of catenanes. A general feature of such “passive template” syntheses,^13^ in which non-covalent interactions pre-organise the covalent subcomponents in a threaded architecture prior to the CuAAC reaction, is that additional functionality must be included in the covalent structure of both components to provide the required pre-organisation. These functional groups remain in the interlocked product and, although the inter-component interactions they often engender can be exploited in the design of molecular machines,^13^ this approach imposes structural limitations on the products available for study.

The active template (AT) approach to interlocked molecules,^14^–^16^ removes the need for such templating units in the sub-components of the interlocked molecule. The potential of this methodology for the synthesis of porphyrin-containing architectures was demonstrated by Anderson and co-workers in the synthesis of diyne-linked porphyrin rotaxanes and catenanes using an AT-Glaser^17^ methodology.^18^ Furthermore, by reducing the size of the macrocycle employed, we have demonstrated that Leigh's active template modification of the CuAAC reaction (AT-CuAAC),^15^ in which a copper centre bound in the cavity of a bipyridine macrocycle mediates the formation of the triazole, is a general approach to functionalised and functional rotaxanes in excellent yield.^19^ Thus, although it has yet to be applied in this context,^20^ the AT-CuAAC reaction appears particularly appropriate for the synthesis of triazole functionalised porphyrin rotaxanes without altering their otherwise desirable properties.

Here we demonstrate the utility of the AT-CuAAC reaction in the synthesis of mechanically interlocked analogues of previously studied triazole-linked porphyrin–corrole conjugates,^21^ and that, because the covalent structure of the chromophores is not altered, photo-induced electron transfer between the tetrapyrrole chromophores is unaffected by mechanical bond formation.^22^ Conversely, the threaded macrocycles significantly modify the steric properties of the system, creating a “mechanical picket fence” motif that suppresses aggregation and ligand driven self-assembly.

## Results and discussion

### Synthesis and characterisation **4**⊂**3**, **5**⊂**3_2_** and **6**⊂**3_4_**

**Scheme 1:**
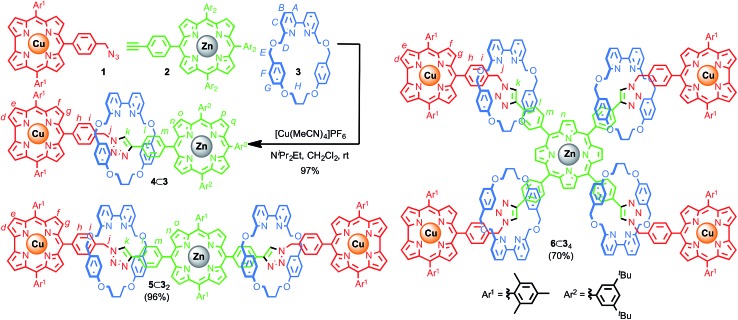
Synthesis of dyad **4**⊂**3** and structures of triad [3]rotaxane **5**⊂**3_2_** and pentad [5]rotaxane **6**⊂**3_4_**.

Porphyrin–corrole diad **4**⊂**3** was synthesised in excellent yield (98% after size exclusion chromatography) by reaction of azide **1**, alkyne **2** and macrocycle **3** in the presence of a Cu^I^ salt (Scheme 1). The extremely high efficiency of the AT-CuAAC reaction allowed this approach to be extended to triad [3]rotaxane **5**⊂**3_2_** and pentad [5]rotaxane **6**⊂**3_4_** in 96% and 70% yield, respectively after size exclusion chromatography, (98% and 91% yield per mechanical bond forming step).

**Fig. 1:**
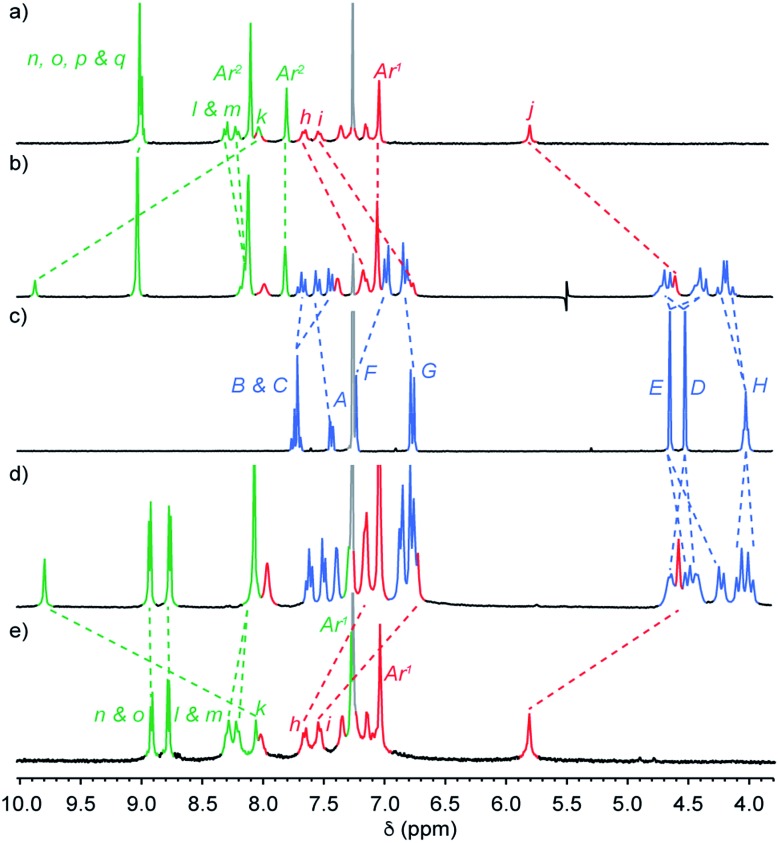
Partial ^1^H NMR (CDCl_3_, 300 MHz, 298 K) of (a) dyad axle **4**, (b) [2]rotaxane **4**⊂**3**, (c) macrocycle **3**, (d) [3]rotaxane **5**⊂**3_2_** and (e) triad axle **5**. Peak assignments as shown in Scheme 1. Residual solvent signals are indicated in light grey.

The mass spectrum (MS) of [2]rotaxane dyad **4**⊂**3** shows a molecular ion at *m*/*z* = 1123.5 consistent with [M + H]^2+^. Comparison of the ^1^H NMR spectra (Fig. 1) of [2]rotaxane **4**⊂**3** with non-interlocked thread **4** and macrocycle **3** further confirmed the formation of the mechanical bond; although many of the resonances associated with the axle remain unaffected by mechanical bond formation (H_n_, H_o_, H_p_, H_q_, and protons associated with Ar^1^ and Ar^2^), which is in keeping with their location away from the threaded region of the axle, triazole proton H_k_ is shifted considerably to lower field (Δ*δ* ∼ 1.8 ppm). This is consistent with previous observations of C–H·N hydrogen bonding between the polarised triazole-H_k_ and the Lewis basic pyridine nitrogen donors^19^ and suggests that the macrocycle is largely localised over the triazole unit. Conversely, benzylic protons H_j_ appear at higher field in the interlocked structure (Δ*δ* ∼ 1.2 ppm) due to the close proximity of the induced magnetic field of the electron rich aromatic units of the macrocycle.

Resonances assigned to the macrocycle component also exhibit the expected changes on mechanical bond formation including the dispersion of bipyridine protons H_A_, H_B_, H_C_, shielding of protons H_F_, and H_G_ of the flanking aromatic units and the splitting of H_D_ and H_E_ into diastereotopic pairs due to the non-centrosymmetric axle desymmetrising the faces of the macrocycle on mechanical bond formation. Analysis of triad [3]rotaxane **5**⊂**3_2_** and pentad [5]rotaxane **6**⊂**3_4_** by MS also confirmed the presence of the corresponding molecular ions (*m*/*z* = 1614.1 [M + H]^2+^ and 1870.7 [M + 3H]^3+^, respectively). Their ^1^H NMR spectra (Fig. 1d and 3a) compared with the non-interlocked components display broadly similar changes to that of **4**⊂**3**.

### Electronic properties of interlocked corrole–porphyrin conjugates **4**⊂**3**, **5**⊂**3_2_** and **6**⊂**3_4_**

Pleasingly, the electronic properties of **4**⊂**3**, **5**⊂**3_2_** and **6**⊂**3_4_** revealed no significant differences compared with the non-interlocked axles.^21^ The interlocked and non-interlocked compounds all display electronic absorption bands at ∼428, 557 and 598 nm associated with the Zn^II^-porphyrin unit, and a band at 413 nm accompanied by broad features between 500 and 660 nm assigned to the Cu^III^-corrole units (Fig. S17†). Furthermore, in all cases the emission associated with the excited singlet state of a ^1^Zn^II^-porphyrin* core was efficiently quenched in the interlocked corrole–porphyrin conjugates.

Femtosecond transient absorption spectroscopy of **4**⊂**3**, **5**⊂**3_2_** and **6**⊂**3_4_** confirmed that, as in the case of the corresponding non-interlocked axles,^21^ the quenching of Zn^II^-porphyrin luminescence is due to efficient and rapid electron transfer from the ^1^Zn^II^-porphyrin* excited state to the Cu^III^-corrole moieties; transient peaks were observed corresponding to the reduced Cu^II^-corrole moiety, and a broad peak appeared corresponding to the Zn^II^-porphyrin˙^+^ radical cation.^23^ Using the transient signal of the Cu^II^-corrole moiety, the rate constant for the charge separation process was evaluated to be *k*_CS_ ∼ 10^11^ s^–1^ for **4**⊂**3**, **5**⊂**3_2_** and **6**⊂**3_4_** with subsequent charge recombination rates of *k*_CR_ = 2.1 × 10^10^ s^–1^, 3.4 × 10^9^ s^–1^, and 3.9 × 10^9^ s^–1^ respectively (*c.f. k*_CS_ = 1.1 × 10^11^ s^–1^ and *k*_CR_ = 5.0 × 10^10^ s^–1^ for **4**).^21^

These results clearly demonstrate that, as proposed, threading of the macrocycles around the arms of the porphyrin core does not significantly affect the electronic properties of the system.

### Effect of threading on the steric properties of pentad **6**⊂**3_4_**

**Fig. 2:**
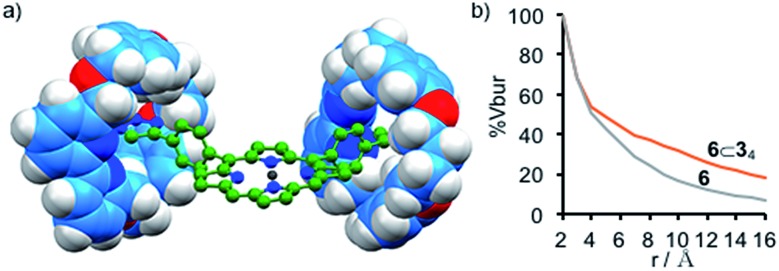
(a) Truncated model (corrole moieties, two arms and axle protons removed for clarity) showing the steric influence of the threaded macrocycles; (b) variation of % *V*_bur_ of a sphere of radius *r* centered on Zn with respect to *r*.

Although the electronic properties of the interlocked products are unchanged compared with the axle moiety, rotaxanes **4**⊂**3**, **5**⊂**3_2_** and **6**⊂**3_4_** clearly have very different steric properties; encircling the triazole moieties with macrocycle **3** significantly increases the steric demand of the linker units. This difference is particularly striking in the case of pentad **6**⊂**3_4_** which lacks sterically bulky aryl groups on the central porphyrin unit; the space filling model of **6**⊂**3_4_** (Fig. 2a) shows that the Zn^II^-porphyrin unit is significantly encumbered by the threaded macrocycles. This steric hindrance was quantified by determining the % buried volume (% *V*_bur_) of spheres centered on the central Zn^II^ ion (Fig. 2b).^24^ At low sphere radii (*r* < 3 Å), the values of % *V*_bur_ for **6** and **6**⊂**3_4_** are identical suggesting that the Zn^II^ center is still accessible to small molecules, as required for catalysis or ligand binding. However, as *r* increases, the values diverge as the threaded macrocycles lead to a higher excluded volume. The comparison between the variation in % *V*_bur_ of **6**⊂**3_4_** and **6** suggests that although the interlocked macrocycles do not affect the accessible volume immediately around the Zn^II^ center, they provide a steric wall at higher radii similar to covalent picket fence porphyrinoids that have been developed for catalytic and photochemical applications.^25^,^26^

An obvious consequence of the difference in steric demand of **6**⊂**3_4_** compared with **6** can be found in their ^1^H NMR spectra; non-interlocked axle **6** displays resonances that are broadened considerably compared with that of [5]rotaxane **6**⊂**3_4_** under the same experimental conditions (Fig. S31 and S32†). This difference is exacerbated as the concentration of the sample is increased; the signals of rotaxane **6**⊂**3_4_** remain sharp while those of non-interlocked axle **6** broaden and shift. The effect of concentration on the ^1^H NMR spectrum of non-interlocked pentad **6** is consistent with aggregation of the Zn^II^-porphyrin unit through π-stacking interactions, as has been widely reported previously.^27^ Conversely, in the case of [5]rotaxane **6**⊂**3_4_**, the macrocycles encircling the four arms of the porphyrin unsurprisingly appear to prevent the close approach of the Zn^II^-porphyrin cores.

To further probe the steric effect of the threaded macrocycles we turned our attention to the well-established ability of the ditopic ligand DABCO (**L**) to direct the formation of [(Zn^II^-porphyrin)_2_**L**] dimers.^29^,^30^ The ^1^H NMR spectra (Fig. S36†) of non-interlocked pentad **6** displayed behaviour consistent with that previously reported as the quantity of **L** was varied: (i) at **L** : **6** ratios up to 0.5 : 1 a signal was observed at –4.92 ppm in the ^1^H NMR spectrum, along with a new signal corresponding to H_n′_ which is consistent with the formation of a [**6**_2_**L**] dimer in slow exchange on the NMR timescale; (ii) once the ratio of **L** : **6** exceeded 0.5 : 1 the signal at –4.92 ppm disappeared and H_n′_ moved to progressively lower field as further ligand was added, stabilising once **L** : **6** = 1 : 1, consistent with fast ligand exchange once excess **L** is present and the formation of monomeric complex [**6L**] in competition with [**6**_2_**L**]. This was further confirmed by cooling the equimolar solution of **6** and **L**; at 273 K a broad signal was observed at –2.96 ppm alongside the reappearance of the signal at –4.92 ppm, consistent with the monomeric species [**6L**] in equilibrium with [**6**_2_**L**] at low temperature. Thus, the speciation of **6** (Fig. 4a) varies as expected with the ratio **L** : **6**. Non-linear regression analysis (see ESI for details†) allowed the association constants for the stepwise association of **6** to ditopic guest DABCO to be determined as *K*_1_ ≥ 5 × 10^6^ M^–1^ and *K*_2_ ≥ 4 × 10^7^ M^–1^, albeit with relatively large associated errors of 30% and 27% respectively.^31^,^32^

**Fig. 3:**
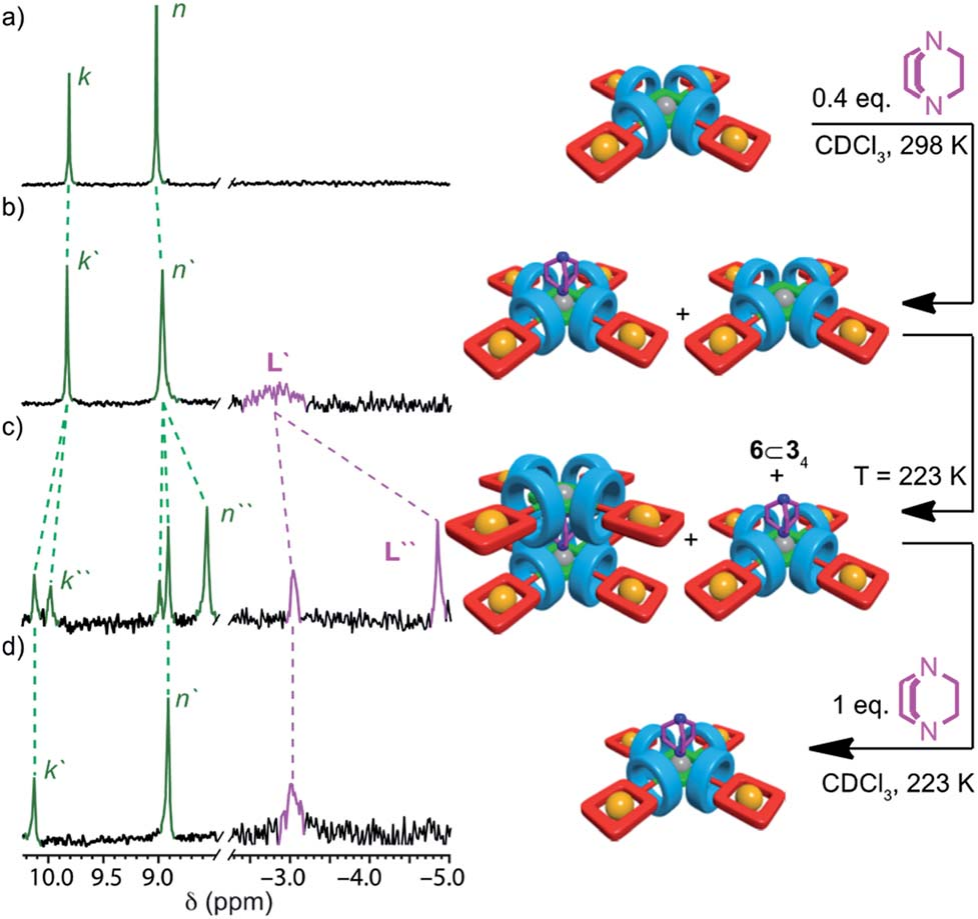
Partial ^1^H NMR (CDCl_3_, 300 MHz) of (a) pentad [5]rotaxane **6**⊂**3_4_** (298 K); (b) **6**⊂**3_4_** + DABCO (0.4 equiv., 298 K); (c) **6**⊂**3_4_** + DABCO (0.4 equiv., 223 K); (d) **6**⊂**3_4_** + DABCO (1 equiv., 223 K). Peak assignments as shown in Scheme 1. Primed (“′”) and doubly primed labels refer to signals attributed to [(**6**⊂**3_4_**)**L**] and [(**6**⊂**3_4_**)_2_**L**] respectively. Cartoon representations have been included to aid clarity but are not intended to be representative of the structures of the complexes formed.^28^

**Fig. 4:**
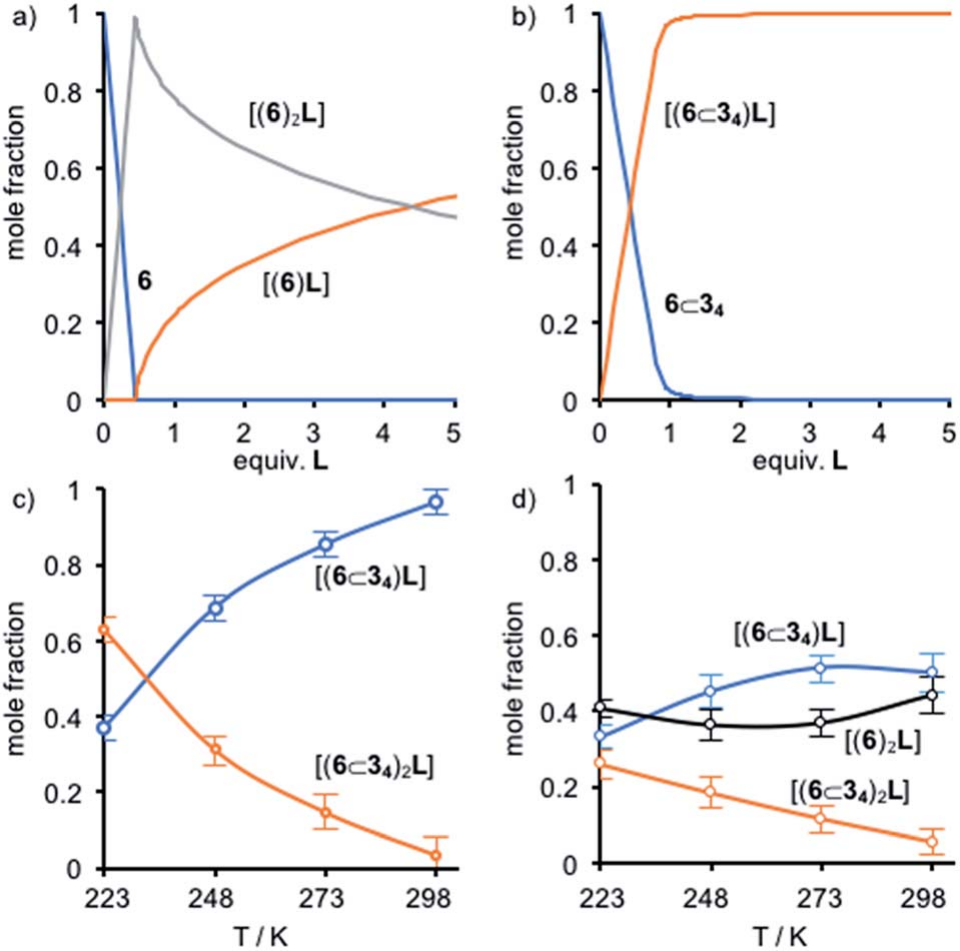
Speciation diagrams for (a) **6** with respect to equiv. **L**; (b) **6**⊂**3_4_** with respect to equiv. **L**; (c) **L** in a **6**⊂**3_4_** + 0.4 **L** mixture with respect to *T*; (d) **L** in a **6** + **6**⊂**3_4_** + **L** (1 : 1 : 1) mixture with respect to *T*.

The behavior of [5]rotaxane **6**⊂**3_4_** is significantly different (Fig. S34†). At 298 K progressive addition of **L** did not lead to the appearance of a signal around –5 ppm corresponding to [(**6**⊂**3_4_**)_2_**L**] but instead the resonances corresponding to triazole proton H_k_ and porphyrin β-protons H_n_ underwent monotonic changes that continued until 1 equivalent of **L** had been added. Alongside these changes, a new broad signal appeared at –2.94 ppm (Fig. 3b) and increased in intensity until 1 equivalent **L** had been added at which point it disappeared. These changes are consistent with the formation of [(**6**⊂**3_4_**)**L**] that undergoes slow exchange with free porphyrin **6**⊂**3_4_** on the ^1^H NMR timescale, progressing to fast exchange once excess **L** is present. Consistent with this, cooling an equimolar mixture of **6**⊂**3_4_** and **L** to 223 K (Fig. 3d) to reduce the rate of ligand exchange resulted in a spectrum consistent with [(**6**⊂**3_4_**)**L**]. Thus, the speciation diagram of **6**⊂**3_4_** with respect to equivalents of **L** (Fig. 4b) is significantly different to that of **6**. Non-linear regression analysis (see ESI for details†) allowed us to determine *K*_1_ to be ≥ 2 × 10^5^ M^–1^ at 298 K.

To further examine this unusual observation, we performed a variable temperature ^1^H NMR study of **6**⊂**3_4_** in the presence of 0.4 equiv. **L** (Fig. S38†). As expected, reducing the temperature to 273 K led a sharpening of the signal at –2.94 ppm, consistent with [(**6**⊂**3_4_**)**L**]. However, surprisingly, a small signal was also observed at –4.62 ppm. Reducing the temperature further to 248 K led to a number of significant changes consistent with the formation of [(**6**⊂**3_4_**)_2_**L**] alongside [(**6**⊂**3_4_**)**L**] and unbound **6**⊂**3_4_**, including the appearance of resonances at –4.62 and 8.6 ppm attributed respectively to **L** and the porphyrin β protons H_n_ of the dimeric complex. Reducing the temperature further to 223 K (Fig. 3c) led to an increase in intensity of signals corresponding to [(**6**⊂**3_4_**)_2_**L**] at the expense of [(**6**⊂**3_4_**)**L**]. Thus, as the temperature is decreased, the mixture of [(**6**⊂**3_4_**)**L**] and **6**⊂**3_4_** present at 298 K is converted to a mixture of [(**6**⊂**3_4_**)_2_**L**], [(**6**⊂**3_4_**)**L**] and **6**⊂**3_4_** until an approximately equimolar mixture of [(**6**⊂**3_4_**)_2_**L**] and [(**6**⊂**3_4_**)**L**] is produced at 223 K (Fig. 4c).

That the [(**6**⊂**3_4_**)_2_**L**] complex forms at all is remarkable given the sterically hindered environment provided by the macrocycles. The effect of temperature suggests that the balance between negative steric interactions and the positive binding interaction between the Zn^II^ center and the N donors of the DABCO ligand are finely balanced. Ultimately, the entropic cost of forming the ternary complex, along with the restrictions to conformational freedom associated with forming such a crowded structure, appear finely balanced against the enthalpic benefit of maximising Zn–N interactions, leading to a strongly temperature dependent self-assembly process.

Finally, we examined the speciation of mixtures of **L**, **6** and **6**⊂**3_4_**. In contrast to previous reports in which mixtures of different Zn^II^-porphyrins in the presence of **L** led to statistical mixtures of dimeric complexes, at low equivalents of **L** there is a high selectivity for formation of [**6**_2_**L**] (Fig. S38†). Furthermore, this selectivity is maintained in a 1 : 1 : 1 mixture of **L**, **6** and **6**⊂**3_4_** as the temperature is varied; **6** is selectively consumed in formation of [**6**_2_**L**] in keeping with the higher stability constant for dimerisation of the non-interlocked axle and we did not observe any evidence of hetero-complex formation. Thus, the mechanical picket fence provided by the macrocycles leads to self-sorting in a mixture of Zn^II^-porphyrin hosts.

## Conclusions

In conclusion, we have demonstrated that the ease and utility of the CuAAC reaction, which has led to its widespread use in the design of functional porphyrinoids for various applications,^2^ is maintained when the active template modification of this reaction is used to produce interlocked analogues. As the covalent structure of the axle is unaffected by mechanical bond formation, the electronic properties of the porphyrin–corrole dyad, triad and pentad reported here are not affected by threading through bipyridine macrocycles, suggesting that the macrocycle provides an alternative, electronically neutral site for structural diversification. Studies comparing the self-assembly behaviour of pentad [5]rotaxane and the corresponding non-interlocked axle component demonstrate that the mechanical bond provides a sterically hindered environment that can modulate intermolecular interactions including π-stacking-driven aggregation and ligand-driven dimerisation. This ability to engineer the steric environment around triazole-functionalised porphyrinoids, an important variable in determining their utility,^25^ without modifying their covalent structure, suggests that such readily available rotaxanes may play a role in the development of novel types of “picket fence” systems. In the longer term, by combining the steric properties demonstrated here with the well-developed chemistry of rotaxane molecular shuttles,^13^ it should be possible to extend these results to produce stimuli responsive systems in which the steric environment around the porphyrin core can be modulated to produce “smart” materials and catalysts.

## Supplementary Material

Supplementary information

Supplementary information

Supplementary information

Supplementary information

Supplementary information

Supplementary information

Supplementary information

Supplementary information

## References

[cit1] Tornøe C. W., Christensen C., Meldal M. (2002). J. Org. Chem..

[cit2] For a recent review on the synthesis and applications of triazole-functionalised porphyrins see: Ladomenou K. Nikolaou V. Charalambidis G. Coutsolelos A. G., Coord. Chem. Rev., 2016, 306 , 1 .

[cit3] Shen D.-M., Liu C., Chen Q.-Y. (2007). Eur. J. Org. Chem..

[cit4] Nierth A., Marletta M. A. (2014). Angew. Chem., Int. Ed..

[cit5] Fazio M. A., Lee O. P., Schuster D. I. (2008). Org. Lett..

[cit6] McDonald A. R., Franssen N., van Klink G. P. M., van Koten G. (2009). J. Organomet. Chem..

[cit7] Maeda C., Kim P., Cho S., Park J. K., Lim J. M., Kim D., Vura-Weis J., Wasielewski M. R., Shinokubo H., Osuka A. (2010). Chem.–Eur. J..

[cit8] Lee C. H., Lee S., Yoon H., Jang W. D. (2011). Chem.–Eur. J..

[cit9] Kolb H. C., Finn M. G., Sharpless K. B. (2001). Angew. Chem., Int. Ed..

[cit10] Hänni K. D., Leigh D. A. (2010). Chem. Soc. Rev..

[cit11] Tuncel D., Cindir N., Koldemir Ü. (2006). J. Inclusion Phenom. Macrocyclic Chem..

[cit12] Kano K., Nishiyabu R., Asada T., Kuroda Y. (2002). J. Am. Chem. Soc..

[cit13] (f) Bruns C. J. and Stoddart J. F., The Nature of the Mechanical Bond: From Molecules to Machines, Wiley, 2016.

[cit14] Crowley J. D., Goldup S. M., Lee A.-L., Leigh D. A., McBurney R. T. (2009). Chem. Soc. Rev..

[cit15] Aucagne V., Hänni K. D., Leigh D. A., Lusby P. J., Walker D. B. (2006). J. Am. Chem. Soc..

[cit16] Lewandowski B., De Bo G., Ward J. W., Papmeyer M., Kuschel S., Aldegunde M. J., Gramlich P. M. E., Heckmann D., Goldup S. M., D'Souza D. M., Fenrandes A. E., Leigh D. A. (2013). Science.

[cit17] Saito S., Takahashi E., Nakazono K. (2006). Org. Lett..

[cit18] Langton M. J., Matichak J. D., Thompson A. L., Anderson H. L. (2011). Chem. Sci..

[cit19] Lahlali H., Jobe K., Watkinson M., Goldup S. M. (2011). Angew. Chem., Int. Ed..

[cit20] During the preparation of this manuscript an example of an AT-CuAAC reaction leading to a threaded strapped porphyrin rotaxane was disclosed: Miyazaki Y. Kahlfuss C. Ogawa A. Matsumoto T. Wytko J. A. Oohora K. Hayashi T. Weiss J., Chem.–Eur. J., 2017, 410.1002/chem.201702553 .

[cit21] Ngo T. H., Zieba D., Webre W. A., Lim G. N., Karr P. A., Kord S., Jin S., Ariga K., Galli M., Goldup S., Hill J. P., D'Souza F. (2016). Chem.–Eur. J..

[cit22] Buston J. E. H., Young J. R., Anderson H. L. (2000). Chem. Commun..

[cit23] In the case of **5**⊂**3_2_** and **6**⊂**3_4_** a transient signal at 690 nm corresponding to ^3^Cu^III^-corrole was also observed due to direct excitation of the corrole unit. See ESI for full details

[cit24] Percent buried volume (% *V*_bur_) is defined as proportion of the volume of a sphere occupied by ligand atoms. It is typically used in the context of catalysis to determine the steric demand of phosphine and N-heterocyclic carbene ligands (A. Poater, B. Cosenza, A. Correa, S. Giudice, F. Ragone, V. Scarano and L. Cavallo, *Eur. J. Inorg. Chem.*, 2009, 1759). Here we use it to compare the steric environments of the interlocked and non-interlocked pentad

[cit25] Collman J. P., Gagne R. R., Reed C., Halbert T. R., Lang G., Robinson W. T. (1975). J. Am. Chem. Soc..

[cit26] It should be noted that aggregation due to π–π stacking is observed by ^1^H NMR at significantly higher concentration (1 to 5 mM) than that at which the absorption and emission spectra are collected (2.5 μM). Thus, the observed suppression of aggregation and the unchanged electronic properties of **6**⊂**3_4_** compared with **6** are consistent with one another

[cit27] Abraham R. J., Evans B., Smith K. M. (1978). Tetrahedron.

[cit28] In particular, it is reasonable to expect that the corrole arms and macrocycles of the porphyrin sub-units will twist relative to and bend away from one another in dimeric complex **6**⊂**3_4_**. Simple molecular modelling suggests this is indeed the case (see ESI)

[cit29] Hunter C. A., Meah M. N., Sanders J. K. M. (1990). J. Am. Chem. Soc..

[cit30] Given that non-interlocked axle **6** exhibits the changes by ^1^H NMR and simple binding equilibrium expected for a Zn^II^-porphyrin in the presence of DABCO, any interaction between the Cu^III^-corrole moiety and DABCO is assumed to be negligible. This is in keeping with the lack, to our knowledge, of any previous report of a DABCO–Cu^III^-corrole complex

[cit31] The errors in the determination of *K*_1_ and *K*_2_ are relatively large due to the limitations of ^1^H NMR analysis. Unfortunately, it was not possible to use UV-vis analysis, which has been shown to be more precise in such measurements, to determine the association constants as the strong absorbance of the corrole units mask the expected changes in the porphyrin Soret band.29a–d

[cit32] With the caveat of the large errors associated with *K*_1_ and *K*_2_, these values suggest a significant degree of positive cooperativity (*α* = 4*K*_2_/*K*_1_ ≈ 15), which is unusual in the DABCO mediated self-assembly of Zn^II^-porphyrin units.29a–d The origin of this effect is uncertain and work is ongoing to establish the cause, but, if it is correct, may be due to interactions between the Cu corrole units that stabilise the dimeric complex

