# Supplementary material for: Porphyrinoid rotaxanes: building a mechanical picket fence
Source: Chem Sci. 2017 Aug 4;8(9):6679–85. doi: 10.1039/c7sc03165c (PMC6103255; doi:10.1039/c7sc03165c)
Supplement: Supplementary file 6 [file SC-008-C7SC03165C-s006.pdf]

Electronic Supplementary Material (ESI) for Chemical Science.
This journal is © The Royal Society of Chemistry 2017

Porphyrinoid Rotaxanes: Building a Mechanical Picket Fence

Thien H. Ngo, Jan Labuta, Gary N. Lim, Whitney A. Webre, Francis D'Souza,* Paul. A. Karr, James E. M. Lewis, Jonathan P. Hill, Katsuhiko Ariga and Stephen M. Goldup**

1. General Experimental Information 2
2. Experimental Procedures 3
3. Discussion of the Characterization of Rotaxane $4\subset3$, $5\subset3_2$, and $6\subset3_4$ 9
4. Photochemical and Electronic Study 14
5. Space Filling Models of $4\subset3$, $5\subset3_2$, $6\subset3_4$, $[(6\subset3_4)L]$ and $[(6\subset3_4)_2L]$ 21
6. % Buried Volume (%Vbur) of Axle 6 and Rotaxane $6\subset3_4$ 24
7. Variable Concentration and Temperature ^{1}H NMR of Axle 6 and [5]rotaxane $6\subset3_4$ 25
8. DABCO Titrations 27
   - Rotaxane $6\subset3_4$ titration with DABCO (L) 27
   - Non-interlocked pentad 6 titration with DABCO (L) 28
   - Complexation preferences in mixed Rotaxane/DABCO/Thread system 30
9. References 32

1. General Experimental Information

Solvents and reagents were obtained from Aldrich Chemicals (Milwaukee, WI), Tokyo Kasei Chemical Co. Ltd. (TCI), Fischer Chemicals (Plano, TX), or Wako Chemical Co. Ltd. NMR spectra were acquired by using a JEOL BX300 spectrometer and chemical shifts (δ) are reported in parts per million (ppm) relative to tetramethylsilane. ESI mass spectra were measured by using a MaXis (Bruker Daltonics, Bremen, Germany) mass spectrometer equipped with a time-of-flight (TOF) analyzer. Mass spectra were measured by using a Shimadzu Axima CFR+ spectrometer using dithranol as the matrix. Azide **1**,[1] acetylene **2**,[1] macrocycle **3,**[2] bis-acetylene porphyrine **S1**[1] and tetra-acetylene porphyrin **S2**[1] were synthesized as previously reported.

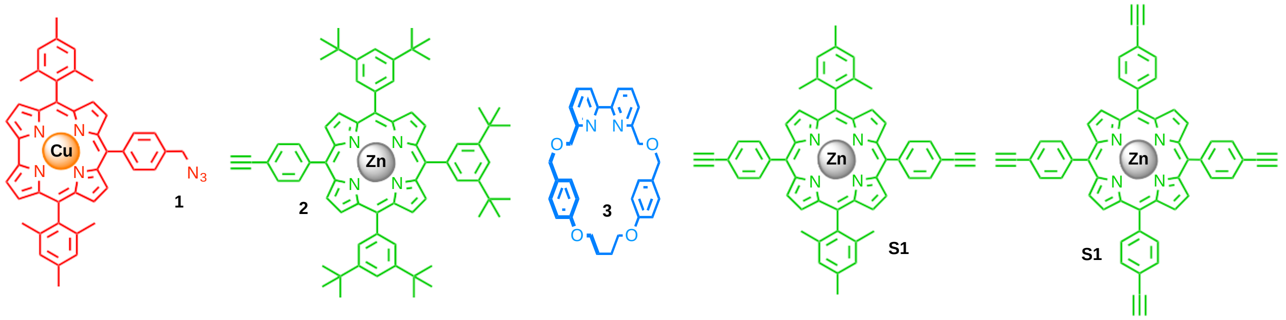

The UV-visible and near-IR spectral measurements were carried out with a Shimadzu 2550 UV-Vis spectrophotometer or Jasco V-670 spectrophotometer. The steady-state fluorescence emission was monitored by using a Varian (Cary Eclipse) Fluorescence Spectrophotometer or a Horiba Jobin Yvon Nanolog spectrofluorimeter equipped with PMT (for UV-visible) and InGaAs (for near-IR) detectors. A right-angle detection method was used for fluorescence measurements at room temperature. All the solutions were purged prior to spectral measurements using argon gas.

Differential pulse voltammetry was recorded on a Princeton Applied Research potentiostat/galvanostat Model 263A using a three electrode system. A platinum button electrode was used as the working electrode, while a platinum wire served as the counter electrode and an Ag/AgCl electrode was used as the reference electrode. Ferrocene/ferrocenium redox couple was used as an internal standard. All the solutions were purged prior to electrochemical measurements with nitrogen gas.

Femtosecond transient absorption spectroscopy experiments were performed using an Ultrafast Femtosecond Laser Source (Libra) by Coherent incorporating diode-pumped, mode locked Ti:Sapphire laser (Vitesse) and diode-pumped intra cavity doubled Nd:YLF laser (Evolution) to generate a compressed laser output of 1.45 W. For optical detection, a Helios transient absorption spectrometer coupled with femtosecond harmonics generator both provided by Ultrafast Systems LLC was used. The source for the pump and probe pulses were derived from the fundamental output of Libra (Compressed output 1.45 W, pulse width 100 fs) at a repetition rate of 1 kHz. 95% of the fundamental output of the laser was introduced into harmonic generator which produces second and third harmonics of 400 and 267 nm besides the fundamental 800 nm for excitation, while the rest of the output was used for generation of white light continuum. In the present study, the second harmonic 400 nm excitation pump was used in all the experiments. Kinetic traces at appropriate wavelengths were assembled from the time-resolved spectral data. Data analysis was performed using Surface Xplorer software supplied by Ultrafast Systems. All measurements were conducted in degassed solutions at 298 K. The estimated error in the reported rate constants is $\pm$10%.

2. Experimental Procedures

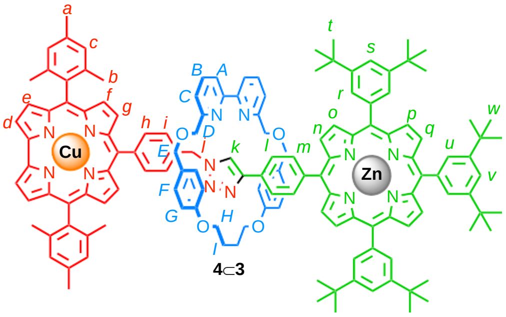

4⊂3

To a solution of porphyrin **2** (26 mg, 2.5 × 10^{-5} mol) and Cu-azidocorrole **1** (18.1 mg, 2.72 × 10^{-5} mol) in 10 mL dichloromethane was added the preprepared mixture of $[Cu(CH_3CN)_4]PF_6$ (8.4 mg, 2.25×10^{-5} mol) and macrocycle **3** (12 mg, 2.5×10^{-5} mol) in 2 mL CH_2Cl. The solution was stirred at room temperature under N_2 atmosphere. A drop of diisopropylethylamine was added. The reaction was monitored using t.l.c. After completion, the reaction mixture was washed with saturated EDTA solution in 10% NH_4OH and brine. The solvent was evaporated under reduced pressure. Purification by size extrusion chromatography (Biobeads SX-1) eluting with chloroform afforded [2]rotaxane **4⊂3** as a brown solid (54 mg, 97%). ^{1}H−NMR (300 MHz, $CDCl_3$, 25°C, TMS): δ=9.87 (s, 1H, H_k), 9.03 (s, 8H, $H_{n,o\ or\ p,q}$), 8.15−8.12 (m, 10H, $H_{l/m}$+$H_{d,e\ or\ f,g}$), 7.99 (br s, 2H, $H_{d,e\ or\ f,g}$), 7.82 (s, 3H, H_s and H_v), 7.71−7.65 (m, 2, H_B), 7.55 (d, ^3J(H,H) = 4.5 Hz, 2H, H_A), 7.44 (d, ^3J(H,H) = 4.5 Hz, 2H, H_C), 7.38 (br s, 2H, $H_{d,e\ or\ f,g}$), 7.16 (d, ^3J(H,H) = 4.5 Hz, 4H, H_c), 7.06 (s, 6H, H_r and H_u), 6.98 (d, 4H, ^3J(H,H) = 5.0 Hz, H_F), 6.84−6.76 (m, 6H, H_G+H_i), 4.73−4.61 (m, 5H, $H_{H/E}$+H_j), 4.44−4.35 (m, 4H, $H_{H/E}$), 4.24−4.313 (m, 4H, H_D), 2.43 (s, 6H, H_a), 2.21−2.19 (m, 2H, H_I), 2.10 (s, 12H, H_b), 1.55 (s, 54H, H_t and H_w) ppm. ^{13}C−NMR (75 MHz, $CDCl_3$, 25°C, TMS): δ 159.5 (C), 159.2 (C), 156.3 (C), 150.5 (C), 150.3 (C), 148.7 (C), 142.0 (C), 137.7 (C), 137.4 (CH), 134.5 (CH), 132.3 (CH), 132.2 (CH), 130.2 (CH), 129.7 (CH), 128.7 (C), 124.4(CH), 128.3 (CH), 124.7 (C), 122.5 (C), 121.5 (C), 120.9 (CH), 115.6 (CH), 77.4 (CH), 73.6 (CH_2), 71.1 (CH2), 67.0 (C), 52.2 (C), 35.2 (C), 31.9 (CH_3), 25.2 (C), 21.4 (C), 20.0 (CH_3) ppm. MS (ESI): calc.: 1123.5 $[M+H]^{2+}$; found: 1123.5 $[M+H]^{2+}$.

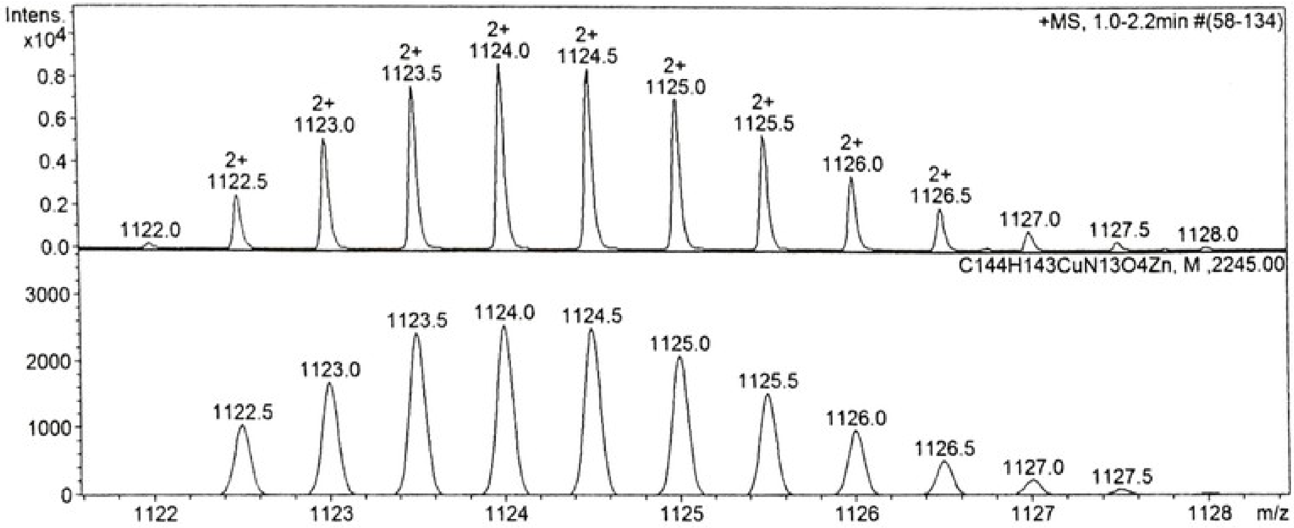

Figure S1: ESI Mass spectrum of [2]rotaxane **4⊂3** (top) and simulated spectrum (bottom).

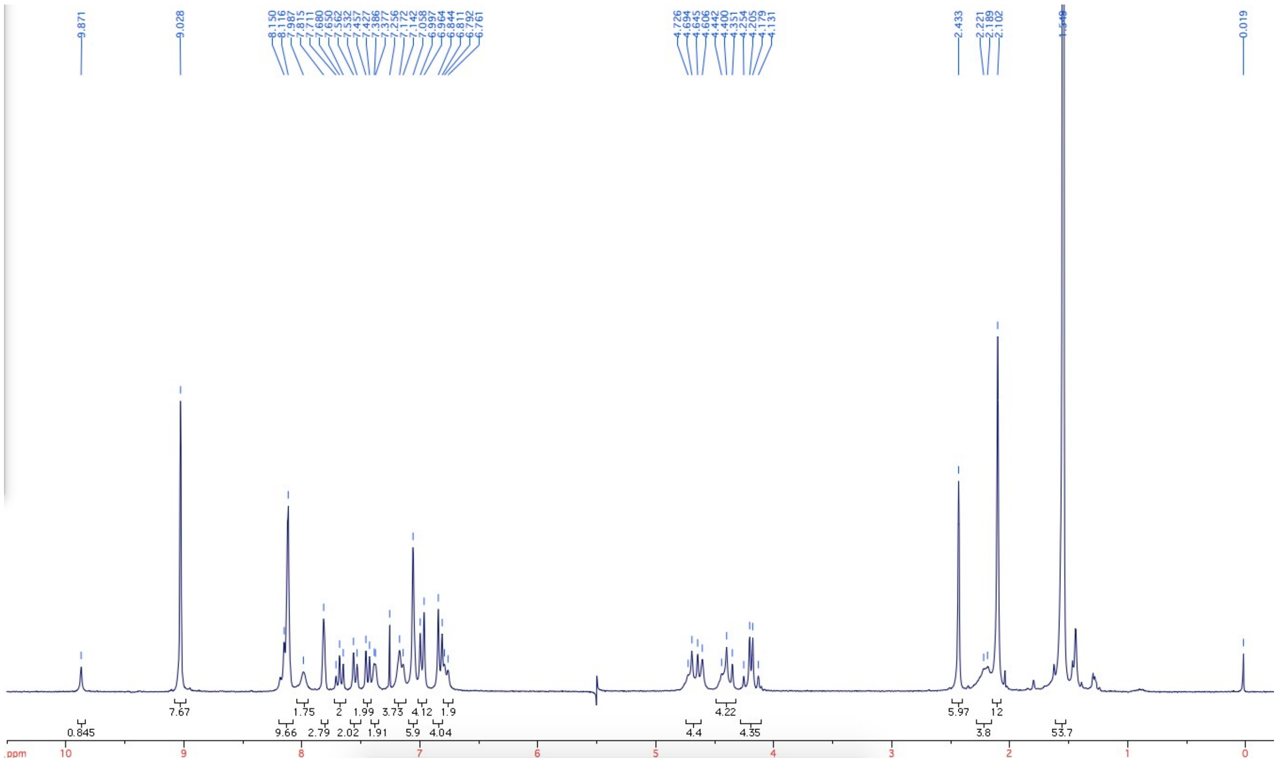
Figure S2: ^{1}H−NMR of [2]rotaxane **4⊂3** in $CDCl_3$ at 25°C

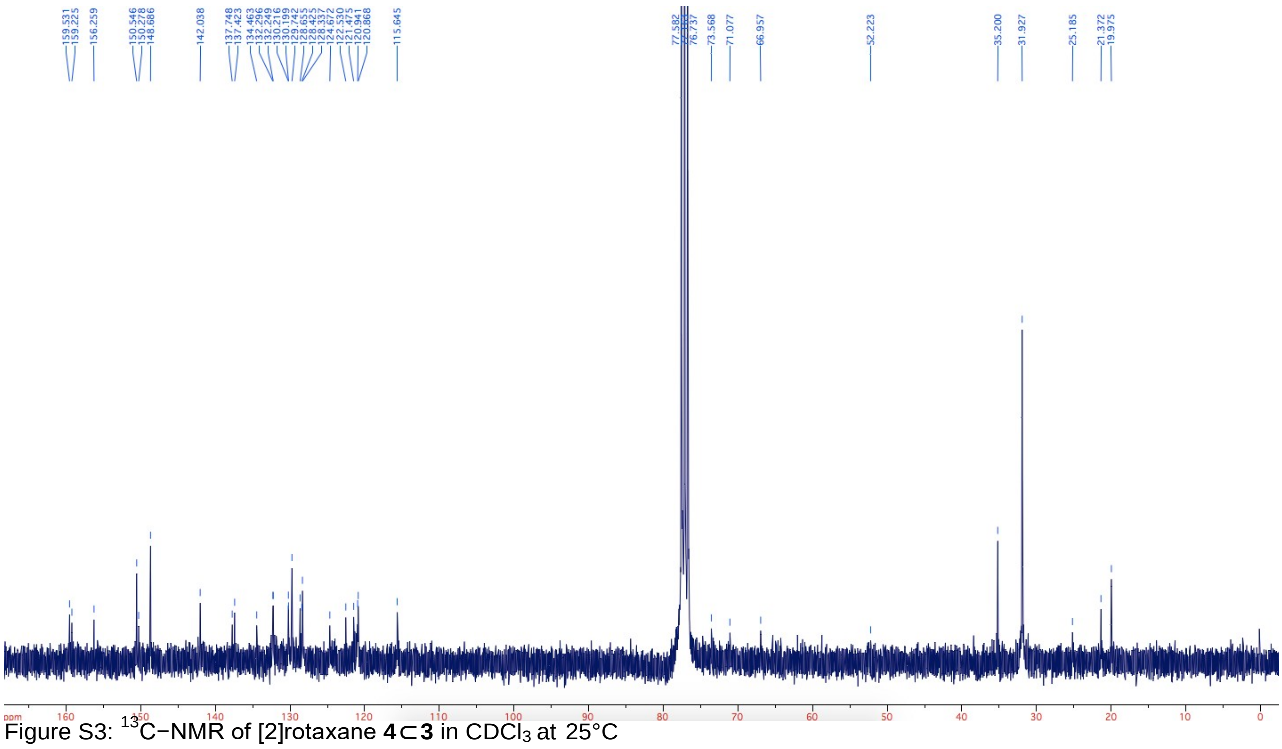
Figure S3: ^{13}C−NMR of [2]rotaxane **4⊂3** in $CDCl_3$ at 25°C

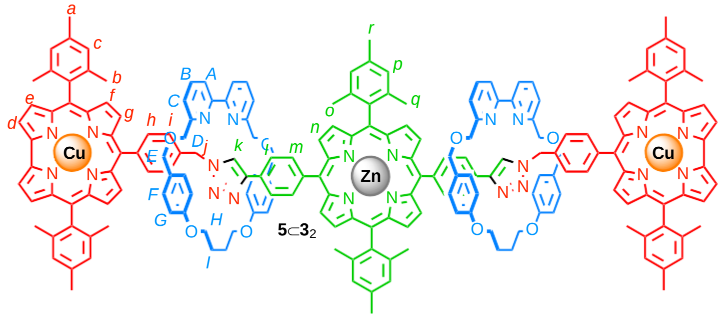

To a solution of bis-acetylene porphyrin **S1** (10.1 mg, 1.25×10^{-5} mol) and copper azidocorrole **1** (18.1 mg, 2.72×10^{-5} mol) in 10 mL CH_2Cl_2 was added the preprepared mixture of $[Cu(CH_3CN)_4]PF_6$ (8.4 mg, 2.25×10^{-5} mol) and macrocycle **3** (12 mg, 2.5×10^{-5} mol) in 2 mL CH_2Cl_2. The solution was stirred at room temperature under N_2 atmosphere. A drop of diisopropylethylamine was added. The reaction was monitored using t.l.c. After completion, the reaction mixture was washed with saturated EDTA solution in 10% NH_4OH and brine. The solvent was evaporated under reduced pressure. Purification by size extrusion chromatography (Biobeads SX-1) eluting with chloroform afforded dirotaxane [3]rotaxane **5⊂3₂** as a brown solid (39 mg, 96%). ^1H−NMR (300 MHz, $CDCl_3$, 25°C, TMS): δ=9.77 (s, 2H, H_k), 8.91 (d, ^3J(H,H) = 4.5 Hz, 2H, $H_{n,o/p,q}$), 8.74 (d, ^3J(H,H) = 4.5 Hz, 2H, $H_{n,o/p,q}$), 8.05 (s, 10H, H_L+H_M), 7.94 (s, 4H, $H_{d,e\ or\ f,g}$), 7.60 (t, ^3J(H,H) = 7.5 Hz, 4H, H_B), 7.48 (d, ^3J(H,H) = 75 Hz, 4H, H_A), 7.37 (s, 6, H_C), 7.27−7.24 (m, 8H, H_h+$H_{d,e\ or\ f,g}$), 7.13 (s_{br}, 8H, $H_{d,e\ or\ f,g}$), 7.03 (s, 12H, H_c and H_p), 6.64 (d, ^3J(H,H) = 8.1 Hz, 8H, H_F), 6.77−6.70 (m, 12H, H_G+H_i), 4.64−4.40 (m, 12H, H_H/H_E+H_j), 4.21 (d, ^3J(H,H) = 60 Hz, 4H, H_H/H_E), 4.08−3.95 (m, 8H, H_D), 2.58 (s, 6H, H_r), 2.41 (s, 12H, H_q), 2.15 (br s, 8H, H_I), 2.08 (s, 24H, H_b), 1.78 (s, 12H, H_a) ppm. ^{13}C−NMR (75 MHz, $CDCl_3$, 25°C, TMS): δ 159.3 (C), 159.0 (C), 156.0 (C), 150.2 (C), 149.9 (C), 149.0 (C), 146.3 (C), 144.5 (C), 143.8 (C), 141.4 (C), 139.3 (C), 139.2 (C), 137.7 (CH), 137.5 (CH), 137.4 (CH), 134.9, 134.4 (CH), 132.4 (C), 130.7 (CH), 130.6 (CH), 130.0 (CH), 128.4, 128.3 (CH), 127.8 (CH), 124.6 (C), 123.9 (CH), 121.3 (CH), 120.7 (CH), 120.4 (C), 119.2 (C), 115.5 (CH), 77.4 (CH), 73.2 (CH_2), 70.7 (CH_2), 66.7 (CH_2), 52.5 (CH_2), 29.9 (CH_2), 25.0 (CH_2), 21.8 (CH_3), 21.6 (CH_3), 21.4 (CH_3), 20.0 (CH_3) ppm. MS (ESI): calc: 1614.1 $[M+H]^{2+}$; found: 1614.1 $[M+H]^{2+}$.

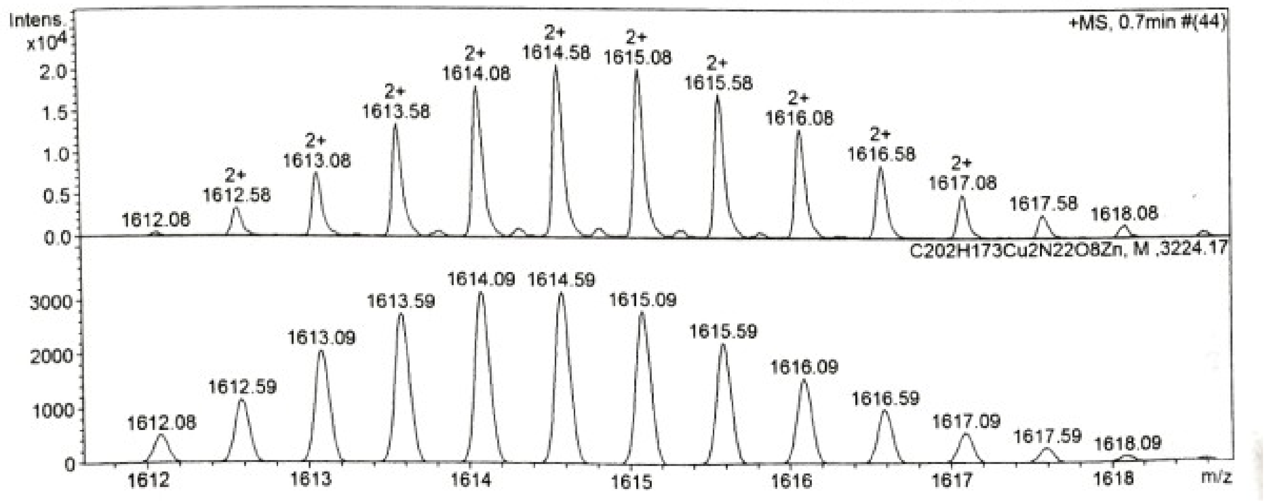

Figure S4: ESI Mass spectrum of [3]rotaxane **5⊂3₂** (top) and simulated spectrum (bottom).

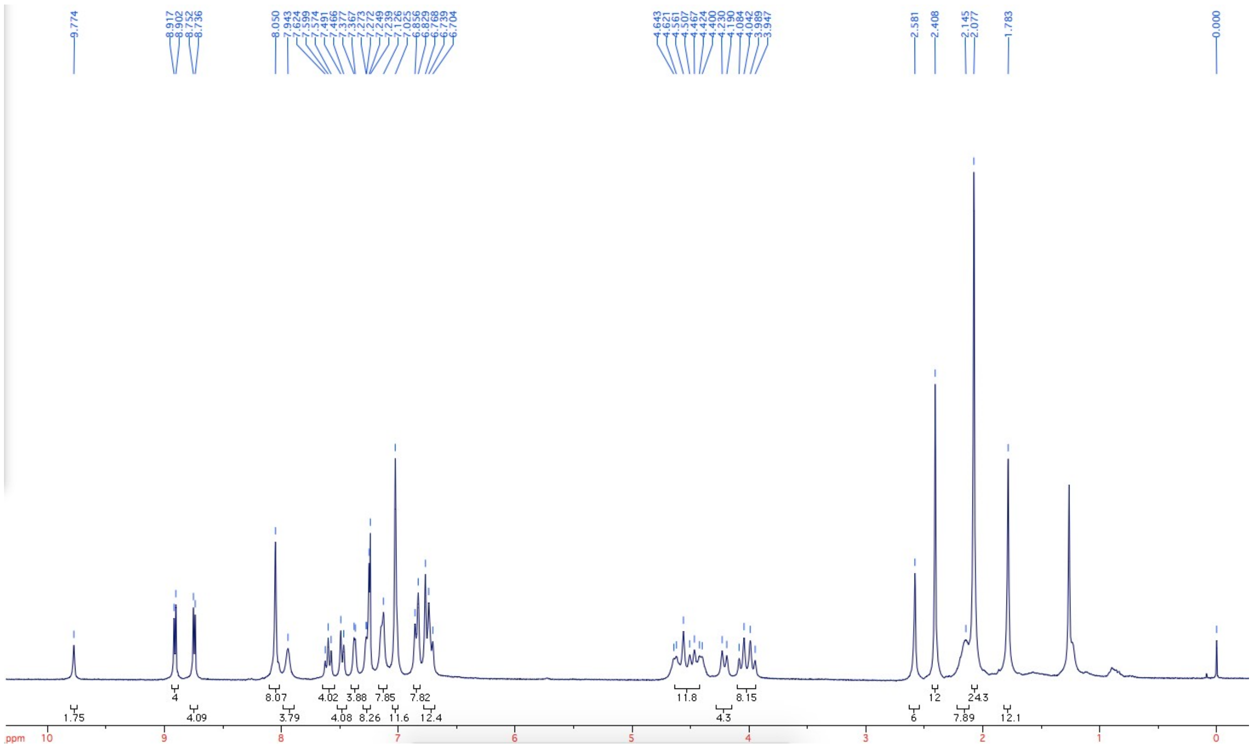

Figure S5: ^{1}H−NMR of [3]rotaxane **5⊂3₂** in $CDCl_3$ at 25°C

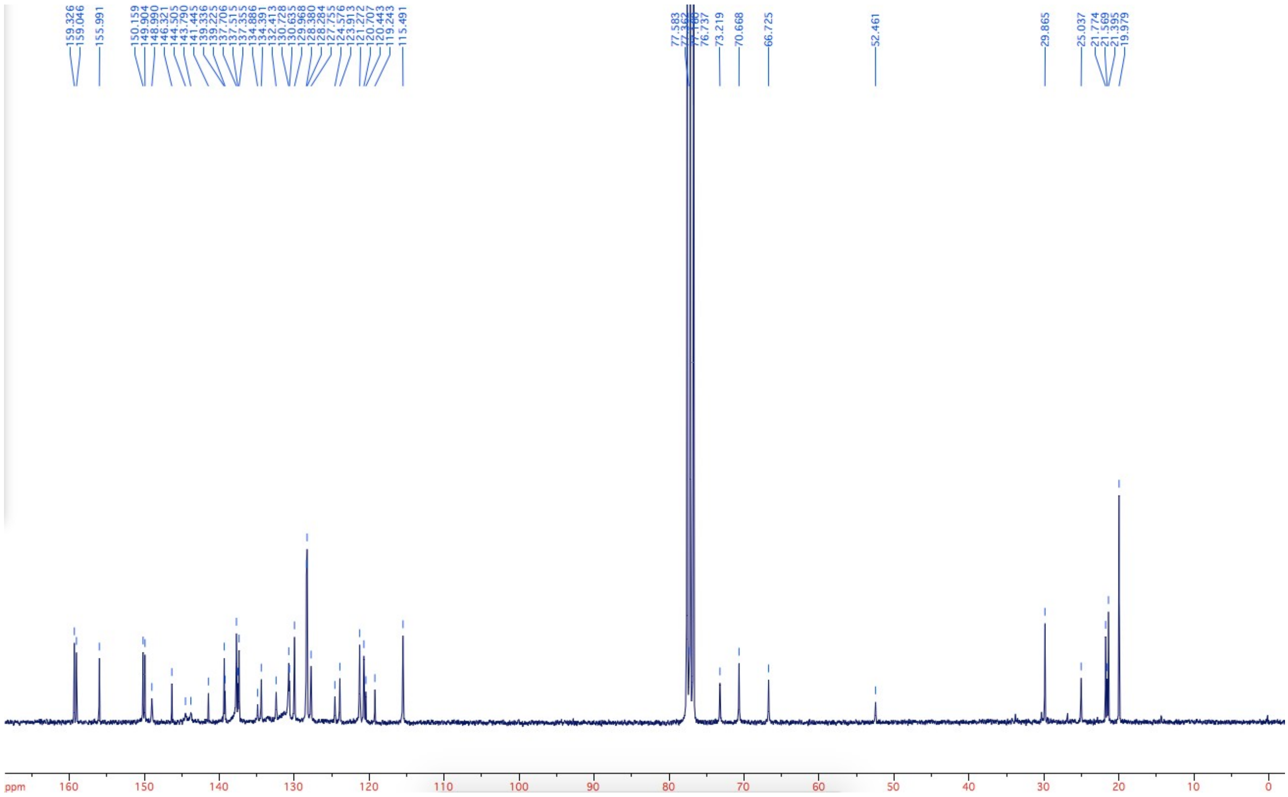

Figure S6: ^{13}C−NMR of [3]rotaxane **5⊂3₂** in $CDCl_3$ at 25°C.

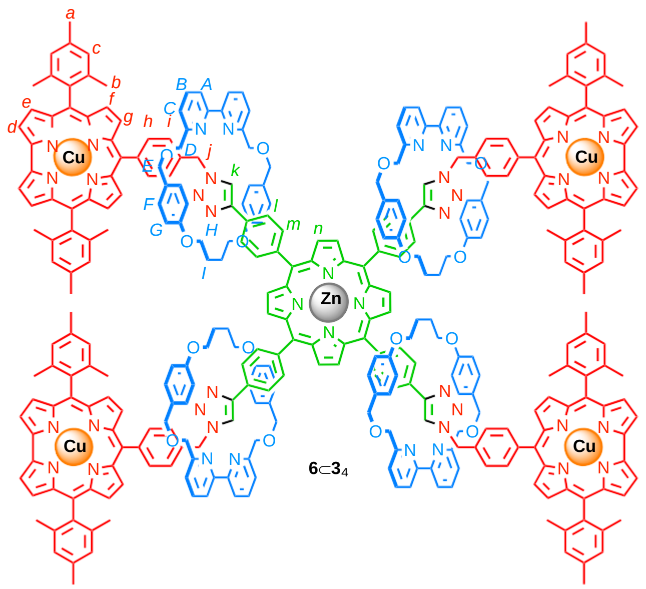

$6\subset3_4$

To a solution of porphyrin tetrakis-(5,10,15,20-(4-acetylenylphenyl))porphyrin (9.65 mg, 1.25×10^{-5} mol) and copper azidocorrole **1** (36.2 mg, 54.2×10^{-5} mol) in 10 mL dichloromethane was added the preprepared mixture of $[Cu(CH_3CN)_4]PF_6$ (16.8 mg, 4.5×10^{-5} mol) and macrocycle **3** (24 mg, 5.0×10^{-5} mol) in 2 mL DCM. The solution was stirred at room temperature under N_2 atmosphere. A drop of diisopropylethylamine was added. The reaction was monitored using t.l.c. After completion, the reaction mixture was washed with saturated EDTA solution in 10% NH_4OH and brine. The solvent was evaporated under reduced pressure. Purification by size extrusion chromatography (Biobeads SX-1) eluting with chloroform afforded [5]rotaxane $\mathbf{6\subset3_4}$ as a brown solid (49 mg, 70%). ^{1}H−NMR (300 MHz, $CDCl_3$, 25°C, TMS): δ=9.85 (s, 4H, H_k), 9.02 (s, 8H, H_n), 8.15−8.05 (m, 16, $H_{l,m}$), 7.94 (s_{br}, 8H, $H_{d,e \text{ or } f,g}$), 7.64 (t, ^3J(H,H) = 7.2 Hz, 8H, H_B), 7.52 (d, ^3J(H,H) = 7.8, 8H, H_A), 7.42(d, ^3J(H,H) = 7.8, 8H, H_C), 7.35 (s_{br}, 8H, $H_{d,e \text{ or } f,g}$), 7.12 (br s, 16, $H_{h+f,g \text{ or } d,e}$), 7.00−6.95 (m, 40H, $H_{f,g \text{ or } d,e}$ and H_c and H_F), 6.82−6.23 (m, 16H, H_G+H_i), 4.67−4.69 (m, 32H, H_H+H_E+H_j), 4.40−4.33 (m, 16H, H_H+H_E), 4.23−4.12 (m, 16H, H_D), 2.38 (s, 24H, H_b), 2.17 (br s_r, 16H, H_I), 2.06 (s, 48H, H_a) ppm. ^{13}C−NMR (75 MHz, $CDCl_3$, 25°C, TMS): δ 159.4 (C), 159.1 (C), 156.1 (C), 150.2 (C), 148.9 (C), 146.2 (C), 144.3 (C), 143.7 (CH), 138.2 (C), 137.7 (C), 137.4 (CH), 134.5 (CH), 132.0 (C), 130.9 (CH), 130.2 (CH), 128.5 (CH), 128.4 (CH), 128.3 (CH), 124.7 (CH), 121.4 (C), 121.3 (CH), 120.8 (CH), 115.5 (CH), 77.4 (CH), 73.4 (CH_2), 70.9 (CH_2), 66.8 (C), 52.5 (C), 29.8 (CH_2), 25.1 (CH_2), 21.3 (CH_3), 19.9 (CH_3) ppm. MS (ESI): calc: 5608.99 [M]; found: 5608.81 [M]; calc.: 1870.7 $[M+3H]^{3+}$; found: 1870.7 $[M+3H]^{3+}$.

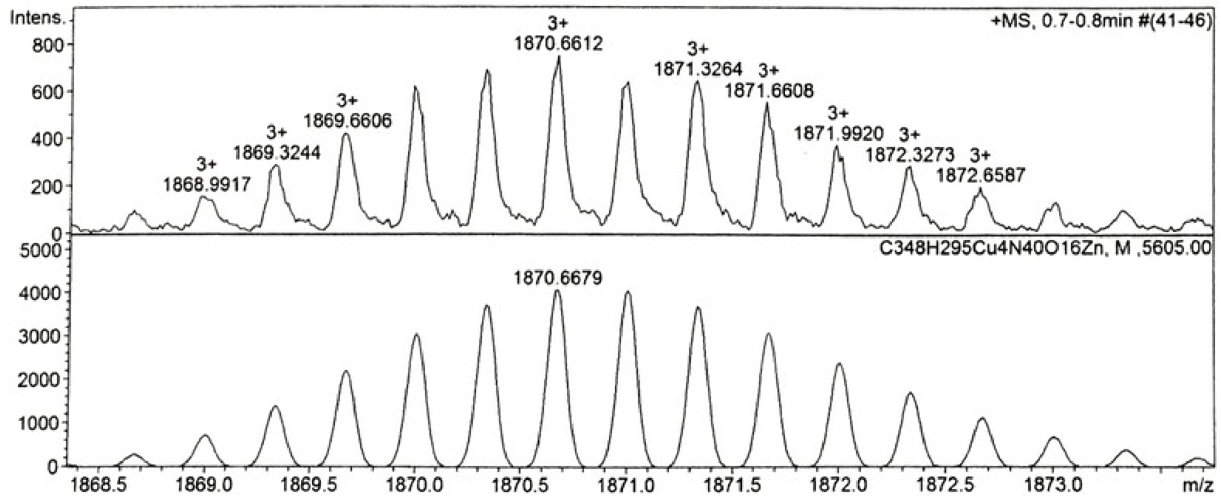

Figure S7: ESI Mass spectrum of [5]rotaxane $\mathbf{6\subset3_4}$ (top) and simulated spectrum (bottom).

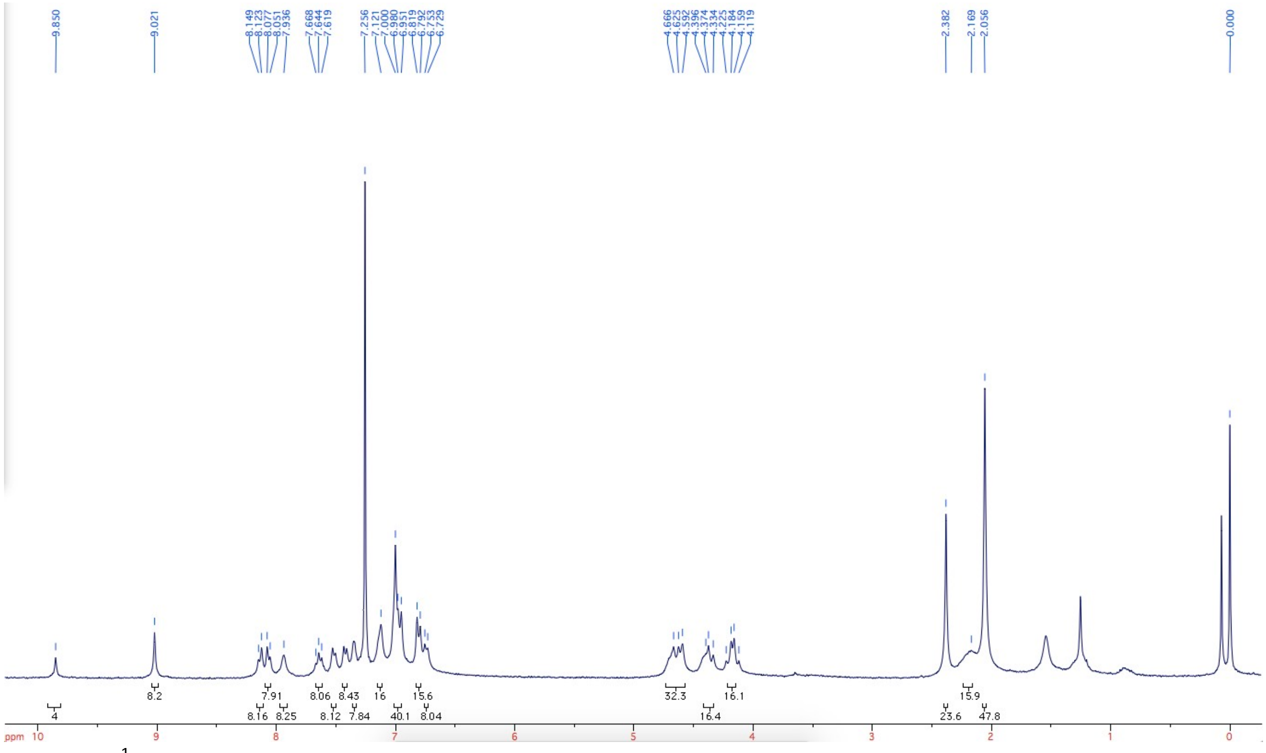

Figure S8: ^{1}H−NMR of [5]rotaxane **6**⊂**3₄** in $CDCl_3$ at 25°C

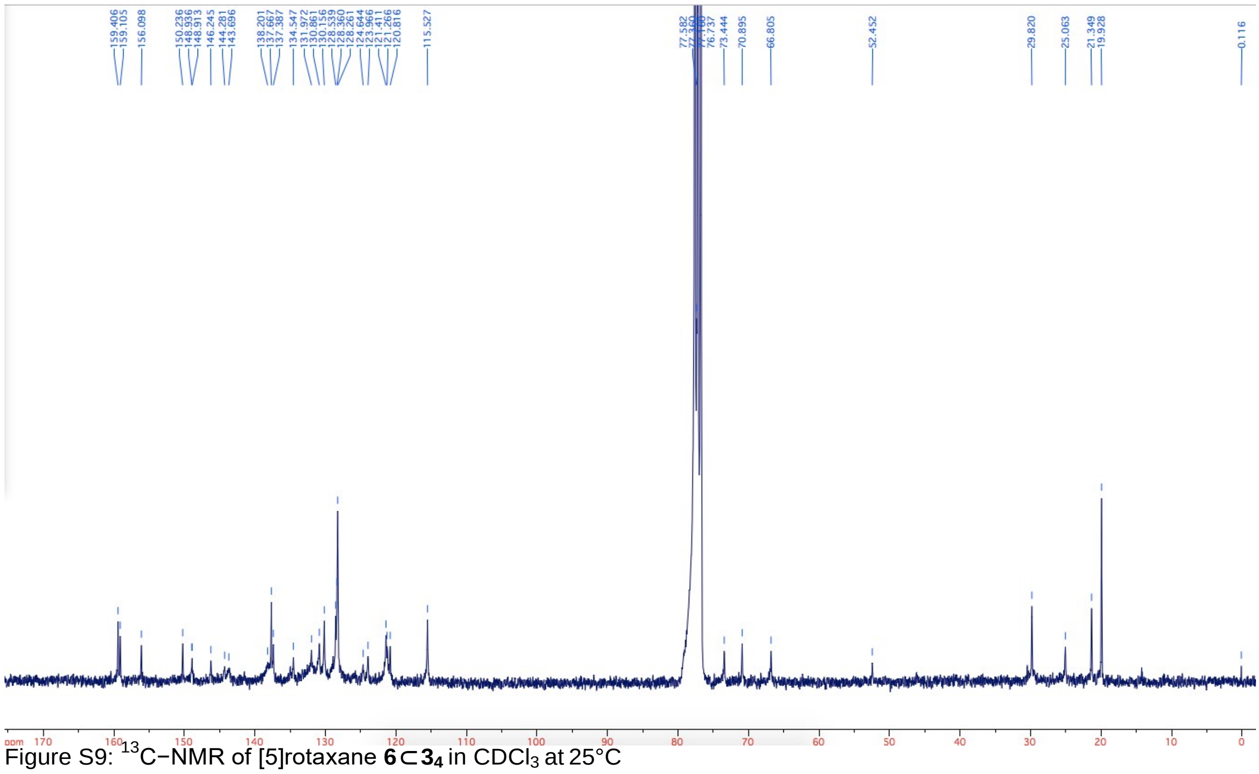

Figure S9: ^{13}C−NMR of [5]rotaxane **6**⊂**3₄** in $CDCl_3$ at 25°C

3. Discussion of the Characterization of Rotaxane 4⊂3, 5⊂3_2, and 6⊂3_4

The mass spectrum (MS) of [2]rotaxane dyad **4⊂3** shows molecular ions at *m/z* = 2246.98 and 1124.5 consistent with $[M+H]^+$ and $[M+2H]^{2+}$ respectively. Comparison of the ^{1}H NMR spectra (Fig 1) of [2]rotaxane **4⊂3** with non-interlocked thread **4** and macrocycle **3** further confirmed the formation of the mechanical bond; although many of the resonances associated with the axle remain unaffected by mechanical bond formation (H_n, H_o, H_p, H_q, and protons associated with Ar^1 and Ar^2), which is in keeping with their location away from the threaded region of the axle, triazole proton H_k is shifted considerably to lower field (Δδ ~ 1.8 ppm). This is consistent with previous observations of C-H-N hydrogen bonding between the polarized triazole-H_k and the Lewis basic pyridine nitrogen donors and suggests that the macrocycle is largely localized over the triazole unit. Conversely, benzylic protons H_j appear at higher field in the interlocked structure (Δδ ~ 1.2 ppm) due to the close proximity of the induced magnetic field of the electron rich aromatic units of the macrocycle. Resonances assigned to the macrocycle component also exhibit the expected changes on mechanical bond formation including the dispersion of bipyridine protons H_A, H_B, H_C, shielding of protons H_F, and H_G of the flanking aromatic units and the splitting of H_D and H_E into diastereotopic pairs due to the non-centrosymmetric axle desymmetrizing the faces of the macrocycle on mechanical bond formation. Analysis of triad [3]rotaxane **5⊂3_2** by MS also confirmed the presence of the expected mass ions (m/z = 3327.16) and the ^{1}H NMR spectrum of **5⊂3_2** (d) displays broadly similar changes to that of **4⊂3** compared with the non-interlocked components.

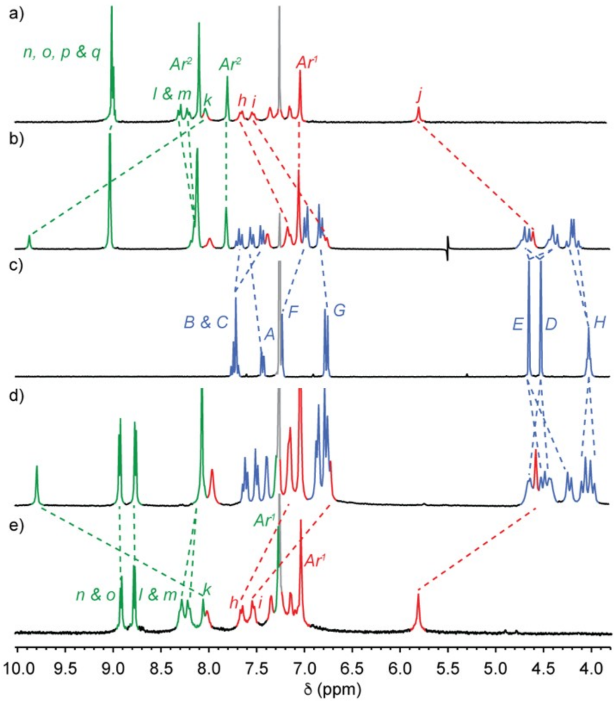

Figure S10. Partial 1H NMR (CDCl3, 300 MHz, 298 K) of a) dyad axle **4**, b) [2]rotaxane **4⊂3**, c) macrocycle **3**, d) [3]rotaxane **5⊂3_2** and e) triad axle **5**. Peak assignments as shown above. Residual solvent signals are indicated in light grey.

MS analysis of [5]rotaxane **6⊂3_4** confirmed the presence of the expected mass ion (*m/z* = 3327.16). In order to assign the spectrum of [5]rotaxane **6⊂3_4** more precisely we have simulated the spectral pattern for each spin system in the molecule (Figure S**12** and Figure

S**13**). This also allows us to decompose the complex spectrum into its subunits as seen in Figure S**13**.

Prior this procedure we have determined which resonances are *J*-coupled using ^{1}H-^{1}H COSY experiment (Figure S**14**, Figure S**15** and Figure S16). There are spin systems which can be fitted as singlets either due to symmetry reasons (Figure S**12**: resonances k, n) or due to very small *J*-coupling constant (Figure S**12**: resonances a, b, c). Spectrum of singlet $S(\delta)$ is fitted using one absorption Lorentzian curve $L(\delta;\ I,\ \delta_0,\ \gamma)$ in the following form:

$$S(\delta)=L(\delta;I,\delta_0,\gamma)=\frac{I\gamma^2}{(\delta-\delta^0)^2+\gamma^2}, \qquad \text{(E1)}$$

where δ (chemical shift; in ppm) is independent variable on abscissa, I is intensity (height) of the corresponding resonance centred at frequency δ_0 (in ppm) and γ (in ppm) is the half-width at half-height.

However most of the spectral patterns consist of AB second order strongly coupled doublet of doublets (see Figure S**12**: e.g. [l, m], [d, e], E, D, etc.). These AB patterns are generated by both geminal and vicinal proton-proton geometries with ^2J and ^3J scalar coupling constants over two and three bonds, respectively. An example of geminal protons is D (or E) resonance and vicinal is [l, m] (or [F, G]) resonance (Figure S**12**). Spectrum of AB pattern $S_{AB}(\delta)$ is fitted using four absorption Lorentzian curves in the following form:

$$\begin{aligned} S_{\mathrm{AB}}(\delta) &= L(\delta;I_{\mathrm{Aout}},\delta_{\mathrm{Aout}},\gamma_{\mathrm{A}})+L(\delta;I_{\mathrm{Ain}},\delta_{\mathrm{Ain}},\gamma_{\mathrm{A}}) \\ &\quad +L(\delta;I_{\mathrm{Bin}},\delta_{\mathrm{Bin}},\gamma_{\mathrm{B}})+L(\delta;I_{\mathrm{Bout}},\delta_{\mathrm{Bout}},\gamma_{\mathrm{B}}), \end{aligned} \qquad \text{(E2)}$$

where $L(\delta;\ I_x,\ \delta_x,\ \gamma_y)$ have the same meaning as in eqn. E1. The AB system obeys following eqns. E3-E10 [Ref. 8] for position of each Lorentzian curve (δ_1, δ_2, δ_3 and δ_4), for intensity relations between outer (I_{Aout}, I_{Bout}) and inner (I_{Ain}, I_{Bin}) resonances of A and B parts, and for half-width at half-height (γ_A, γ_B) of A and B parts:

$$\delta_{\mathrm{Aout}}=\tfrac{1}{2}(\delta_{\mathrm{A}}+\delta_{\mathrm{B}}+Q+J_{\mathrm{AB}}), \qquad \text{(E3)}$$

$$\delta_{\mathrm{Ain}}=\tfrac{1}{2}(\delta_{\mathrm{A}}+\delta_{\mathrm{B}}+Q-J_{\mathrm{AB}}), \qquad \text{(E4)}$$

$$\delta_{\mathrm{Bin}}=\tfrac{1}{2}(\delta_{\mathrm{A}}+\delta_{\mathrm{B}}-Q+J_{\mathrm{AB}}), \qquad \text{(E5)}$$

$$\delta_{\mathrm{Bout}}=\tfrac{1}{2}(\delta_{\mathrm{A}}+\delta_{\mathrm{B}}-Q-J_{\mathrm{AB}}), \qquad \text{(E6)}$$

$$Q=\sqrt{(\delta_{\mathrm{A}}-\delta_{\mathrm{B}})^2+J_{\mathrm{AB}}^2}, \qquad \text{(E7)}$$

$$I_{\mathrm{Aout}}=I_{\mathrm{Ain}}\frac{Q-J_{\mathrm{AB}}}{Q+J_{\mathrm{AB}}}, \qquad \text{(E8)}$$

$$I_{\mathrm{Bout}}=I_{\mathrm{Bin}}\frac{Q-J_{\mathrm{AB}}}{Q+J_{\mathrm{AB}}}, \qquad \text{(E9)}$$

$$\gamma_{\mathrm{B}}=\gamma_{\mathrm{A}}\frac{I_{\mathrm{Ain}}}{I_{\mathrm{Bin}}}. \qquad \text{(E10)}$$

Where δ_A and δ_B are true resonance frequencies of A and B spins, respectively, and J is scalar coupling constant between them. In these equations we also consider different relaxation rates for A and B spins which is reflected by possibility of having different values of γ_A and γ_B while keeping the integral area of both resonances the same. All variables are in ppm units and can be converted to Hz units using relation ν (in Hz) = δ (in ppm) $\times\ \nu_0$,

where ν_0 is the operating frequency of the spectrometer (in MHz); in the present case 300.4 MHz. Independent and fitted parameters are: δ_A, δ_B, J, I_{Ain}, I_{Bin} and γ_A.

Graphical representation of eqns. E2-E10 can be seen in Figure S**11**a. Some spin systems (such as resonances H in Figure S**12**) are first order extensions to AB system as schematically seen in Figure S**11**b. In this case the spin system is treated as ABXY with second order AB structure (J_{AB} coupling constant) and first order AX, AY, BX and BY interactions (J_{AX}, J_{AY}, J_{BX} and J_{BY} coupling constants). Some other spin system such as resonances A, B and C (Figure S**12**) are treated as ABC spin system with strong J_{AB} and J_{BC} constants and weak J_{AC} coupling constant.

When each spectral pattern was roughly placed on top of experimental spectrum nonlinear least square fitting method was used to precisely fit whole spectrum (with linear function as an baseline).

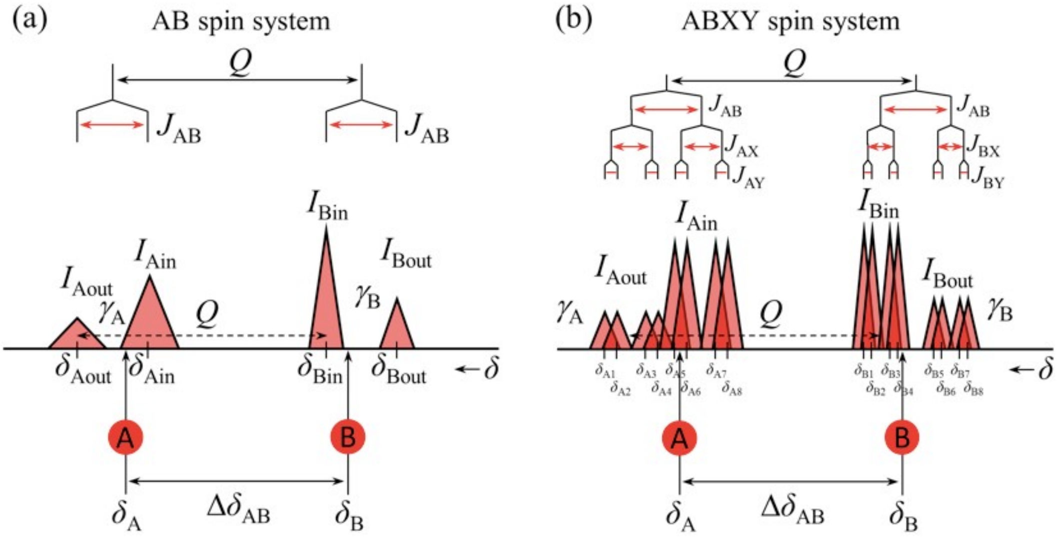

Figure S11: (a) Model of AB second order strongly coupled doublet of doublets (4 Lorentzians) with scalar coupling constant J_{AB}. (b) Model of ABXY spin system (16 Lorentzians) with second order J_{AB} and first order J_{AX}, J_{AY}, J_{BX} and J_{BY} coupling constants. ABXY model is simple extension of AB model using two additional splitting J_{AX} and J_{AY} for A resonance and J_{BX} and J_{BY} for B resonance, respectively.

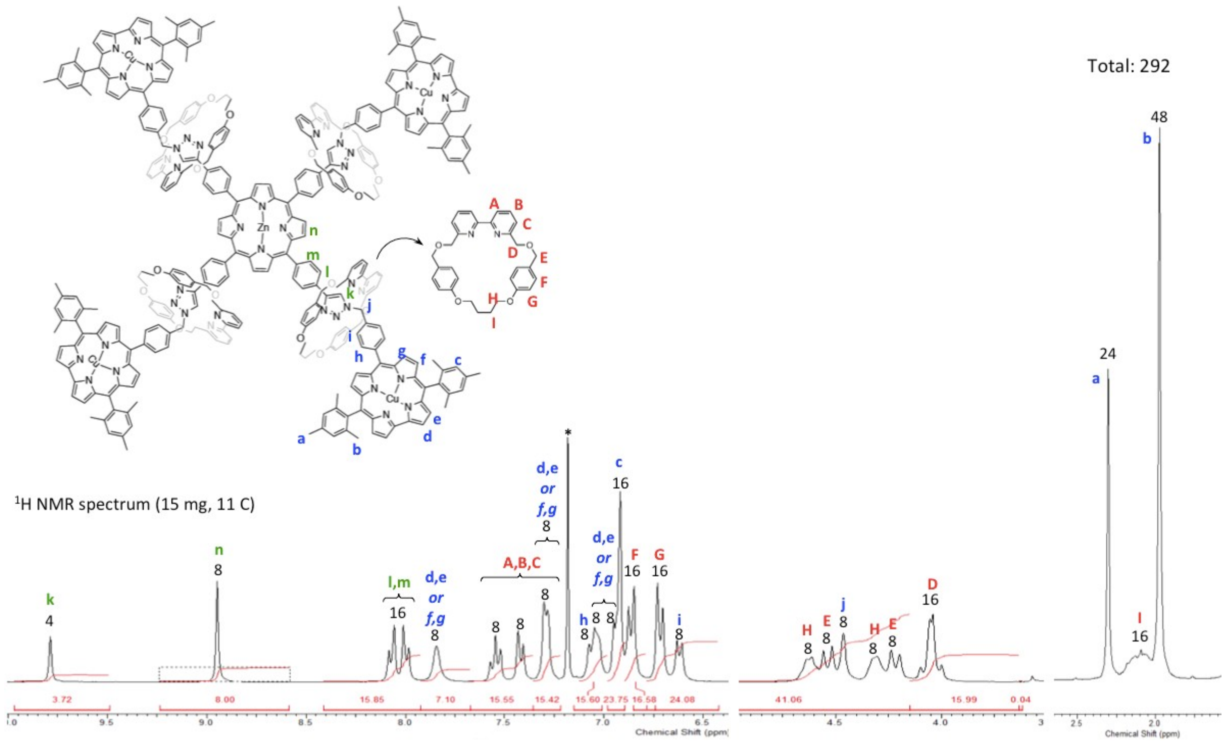

Figure S12: Peak assignment of on ^{1}H NMR spectrum (300 MHz) of [5]rotaxane **6**$\subset$**3**$_4$ in $CDCl_3$ at 11°C based on COSY experiments in Figure S14, Figure S15 and **Figure S16** and above described fitting procedure.

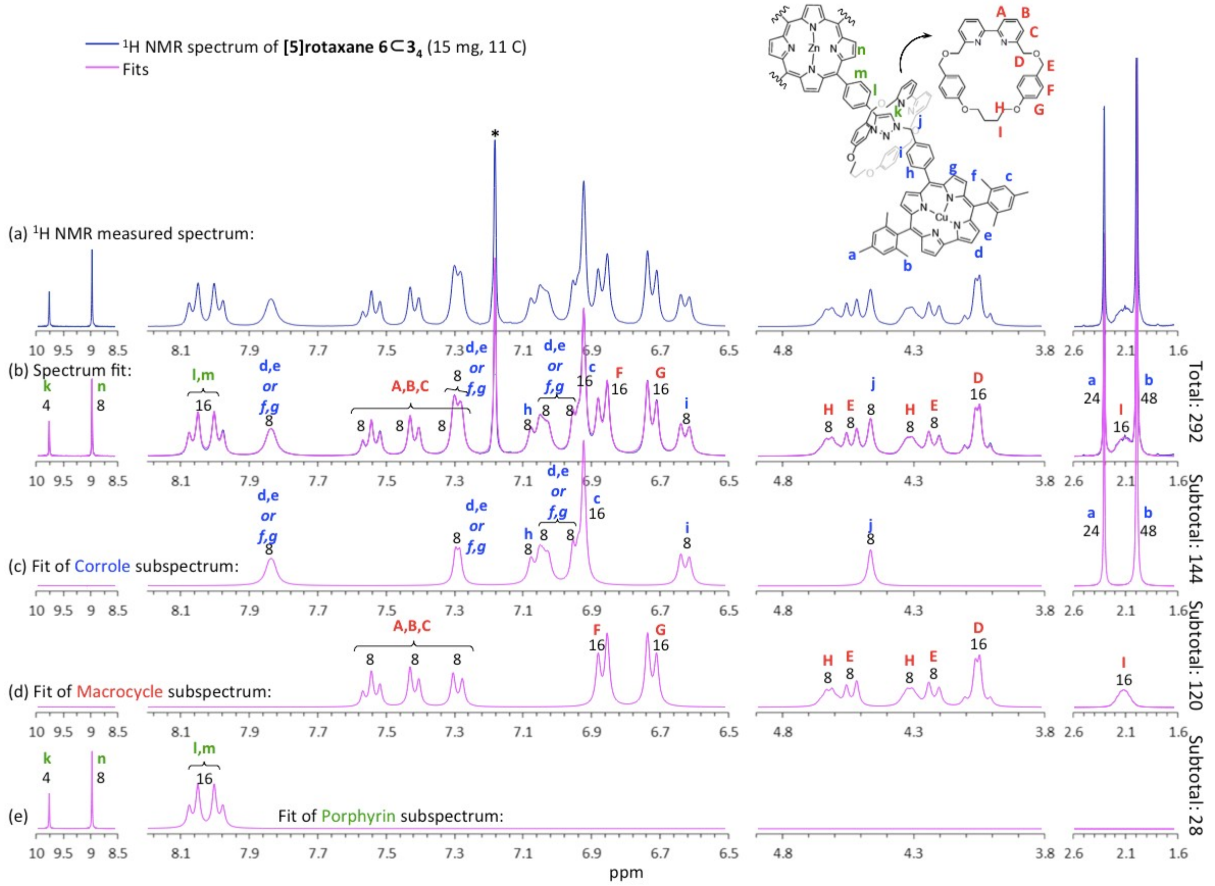

Figure S13: Peak assignment and fitting of each sub-unit of ^{1}H NMR spectrum (300 MHz) of [5]rotaxane **6**$\subset$**3**$_4$ in $CDCl_3$ at 11°C. Nonlinear least square fitting method was used with appropriate spectral pattern corresponding to each spin system. Asterisk denotes residual $CHCl_3$. (a) Spectrum of [5]rotaxane **6**$\subset$**3**$_4$ as measured (blue line). (b) Fitted spectrum (magenta line) on top of measured spectrum (blue line). Note that there is almost identical match. (c), (d) and (e) Sub-spectra of [5]rotaxane **6**$\subset$**3**$_4$ as extracted from the whole fit shown in (b).

[5]rotaxane 6⊂3$_4$

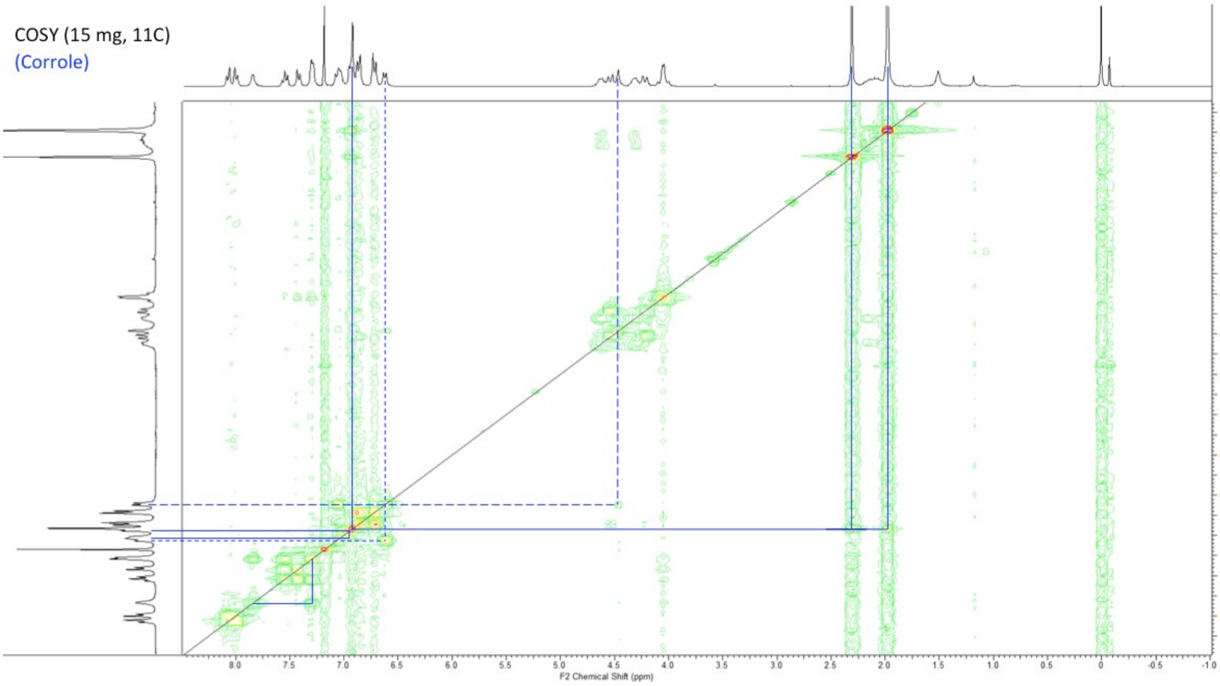

Figure S14: ^{1}H-^{1}H COSY spectrum (300 MHz) of macrocycle unit of [5]rotaxane **6**⊂**3**$_4$ in $CDCl_3$ at 11°C

[5]rotaxane 6⊂3$_4$

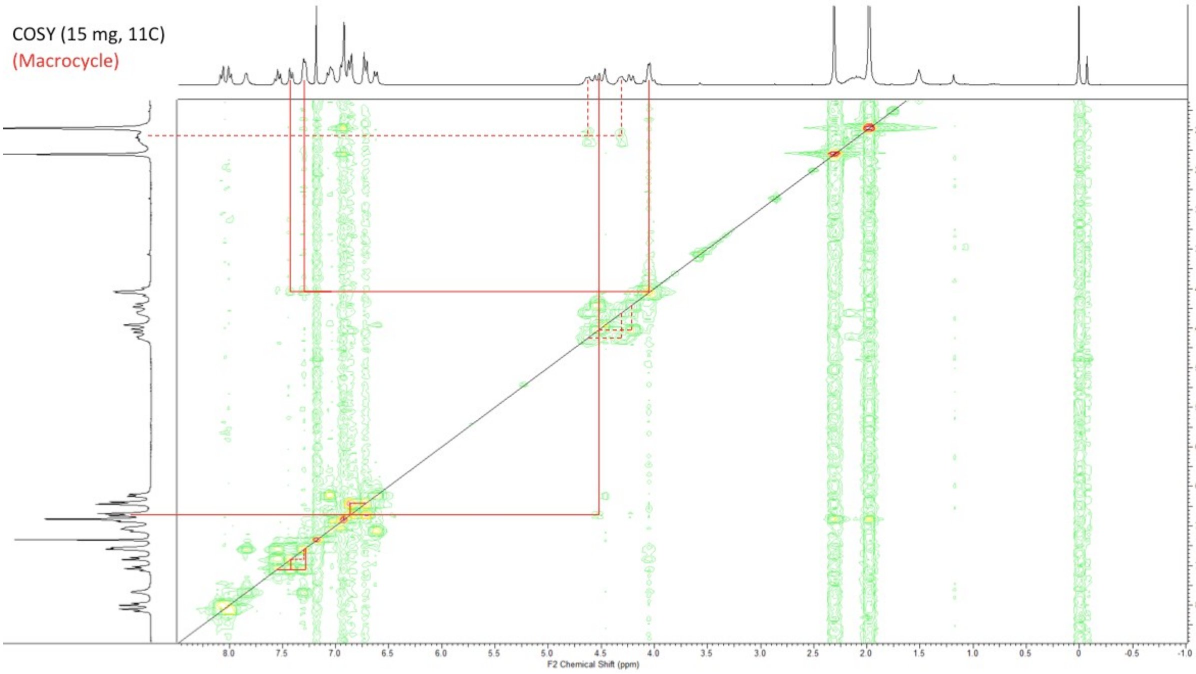

Figure S15: ^{1}H-^{1}H COSY spectrum (300 MHz) of macrocycle unit of [5]rotaxane **6**⊂**3**$_4$ in $CDCl_3$ at 11°C

[5]rotaxane $6\subset3_4$

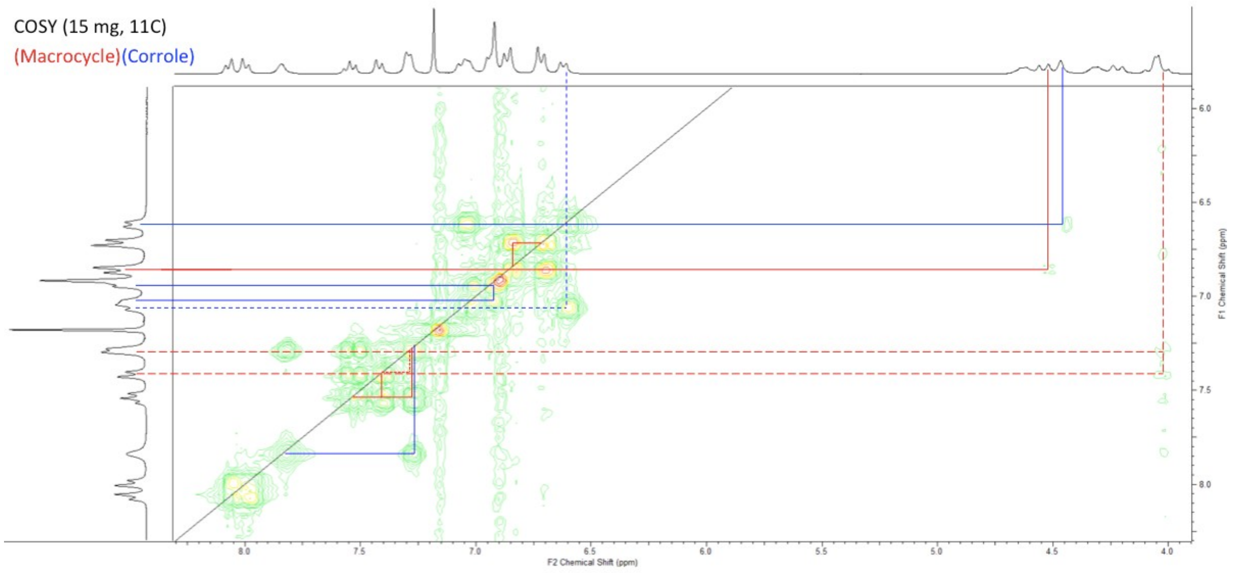

Figure S16: ^1H-^1H COSY spectrum (300 MHz) of corrole and macrocycle units of [5]rotaxane $6\subset3_4$ in $CDCl_3$ at 11°C

4. Photochemical and Electronic Study

Examination of the electronic properties of **4⊂3**, **5⊂3**$_2$ and **6⊂3$_4$**, alongside control compounds Zn-porphyrin (ZnP) and Cu-corrole (CuC), revealed little difference with the non-interlocked axles;[1] the interlocked and non-interlocked compounds (Figure S**17**c,d) all display electronic absorption bands at ~428, 557 and 598 nm associated with the Zn-porphyrin unit (Figure S**17**a), and bands at 413 nm accompanied by broad features in the visible range between 500 nm and 660 nm assigned to the Cu-corrole units (Figure S**17**b). Furthermore, in all cases the emission associated with the excited singlet state of the Zn-porphyrin core was efficiently quenched by the Cu-corrole moiety (Figure S**17**d) suggesting occurrence of excited state events from the singlet-excited state of zinc porphyrin (^{1}ZnP*).

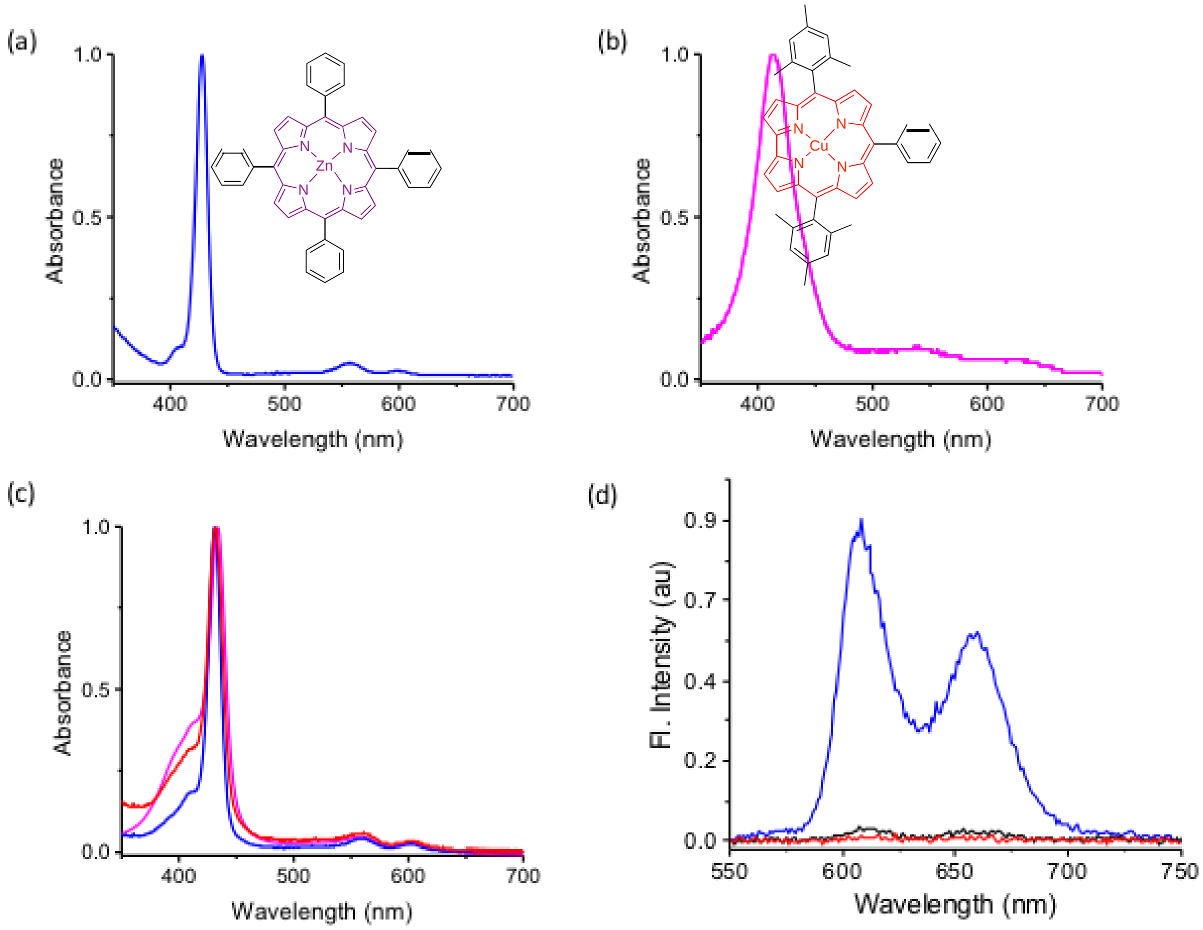

Figure S17: (a) Absorbance (normalized) of (a) ZnP; (b) CuC; (c) $\mathbf{4}\subset\mathbf{3}$ (blue), $\mathbf{5}\subset\mathbf{3}_2$ (red) and $\mathbf{6}\subset\mathbf{3}_4$ (magenta); and (d) fluorescence (excitation at 427 nm) of ZnP (blue), $\mathbf{4}\subset\mathbf{3}$ (black) and $\mathbf{5}\subset\mathbf{3}_2$ (red). All concentrations were held at 2.5×10^{-6} M in benzonitrile. No detectable fluorescence was observed for $\mathbf{6}\subset\mathbf{3}_4$.

The molecular electrostatic potential (MEP) maps with the optimized structures on the Born-Oppenheimer surface in the background, optimized at the B3LYP/6-31G*[3] level are shown in Figure S**18**. The presence of rotaxane introduced additional rigidity confining the donor-acceptor distance and orientation. The Cu-Zn distance in the optimized geometries were approximately 18 Å, the edge-to-edge distances were approximately 11 Å, the Cu-triazole center was approximately 11 Å, and the Zn-triazole center was approximately 11 Å. To a large extent, the ZnP and CuC entities were in the same plane. The MEP maps suggested the expected electron rich site of ZnP and electron poor site of CuC. The electron rich nature of the rotaxane bridges was also apparent.

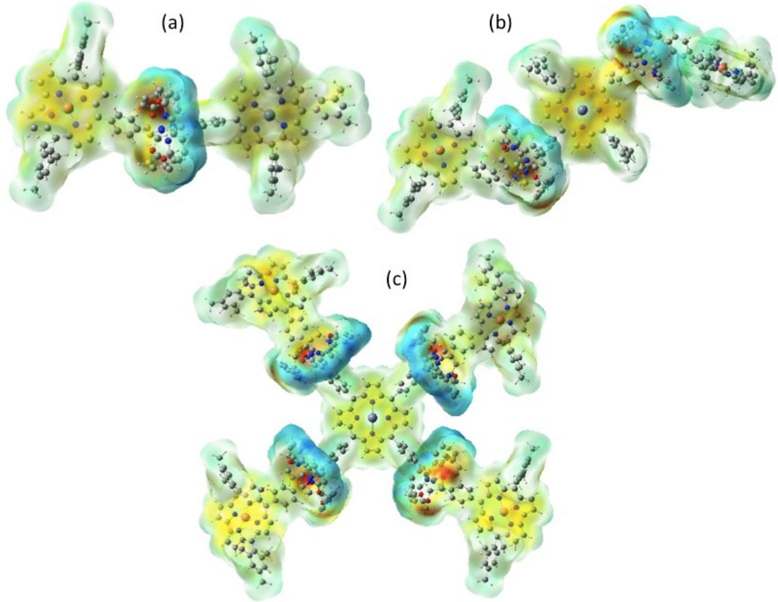

Figure S18: Molecular electrostatic potential (MEP) maps of **4⊂3**, **5⊂3₂** and **6⊂3₄** from B3LYP/6-31G* optimizations.

The first oxidation and first reduction of control ZnP and CuC compounds were located at 0.32 and -1.79, and at 0.28 and -0.61 V vs. Fc/Fc^+, respectively. As shown in Figure S**19** the first reduction in the cases of **4⊂3**, **5⊂3₂** and **6⊂3₄** was located at -0.60 while the oxidation (overlap of first oxidations of ZnP and CuC entities) was at 0.31 V vs. Fc/Fc^+.

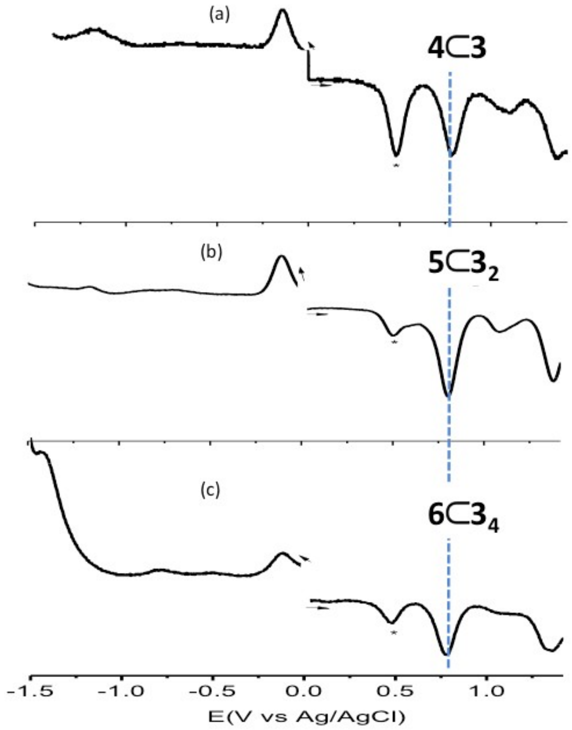

Figure S19: DPVs of (a) **4⊂3**, (b) **5⊂3₂** and (c) **6⊂3₄** in benzonitrile, 0.1 M (n-Bu_4N)ClO_4. Scan rate = 5 mV/s, pulse width = 0.25 s, pulse height = 0.025 V. The oxidation wave of ferrocene used as the internal reference is shown with an asterisk.

It is important to note that the presence of rotaxane rings in **4⊂3**, **5⊂3₂** and **6⊂3₄** did not perturb the redox potentials appreciably, especially that of CuC known for its facile reduction potential.[1] Further, using the Rehm-Weller approach,[4,5] free-energy calculations for charge-recombination (ΔG_{CR}) and charge-separation from the singlet excited zinc porphyrin (ΔG_{CS}) processes were estimated, and an energy level diagram was established (Figure S**20**). These calculations revealed values of ΔG_{CS} = -1.20 eV and ΔG_{CR} = -0.84 eV in

benzonitrile. The large exothermic ΔG_{CS} value suggests photoinduced electron transfer to be thermodynamically possible in these novel rotaxane connected donor-acceptor systems.

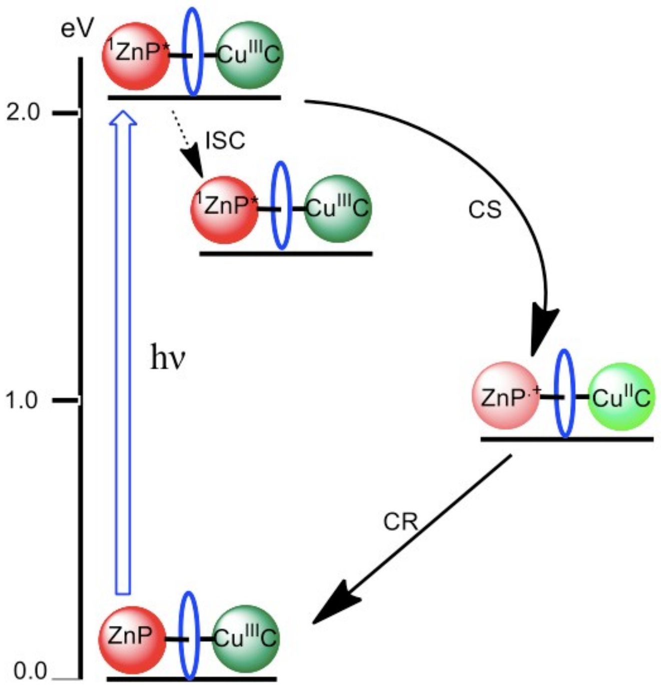

Figure S20: Energy level diagram showing the different photochemical events occurring in rotaxane bridged ZnP-CuC systems in benzonitrile. Energies of different states were evaluated from spectral and electrochemical studies. Solid arrows indicate major photo-processes, dashed arrow indicates minor photo process. ISC = intersystem crossing, CS = charge separation and CR = charge recombination.

Femtosecond transient absorption spectral studies were performed to secure evidence for the occurrence of photoinduced electron transfer. However, to help interpret the transient spectral signals of electron transfer products, spectroelectrochemical studies were performed to characterize the one-electron oxidized donor, zinc porphyrin and the one-electron reduced acceptor, copper corrole. Figure S**21** shows the spectral changes during first oxidation of ZnP in benzonitrile (potential applied = 0.9 V vs. Ag/AgCl). Peaks located at 426, 556 revealed diminished intensity with the appearance of new peaks at 370, 411, 454, and 652 nm. Isosbestic points at 416 and 437 nm were observed. During the first reduction of CuC (applied potential = -0.3 V vs. Ag/AgCl), peaks of the neutral compound located at 414 nm was red-shifted to 435 nm with the appearance of new peaks at 538, 577 and 610 nm. Isosbestic points at 357, and 462 nm were observed.

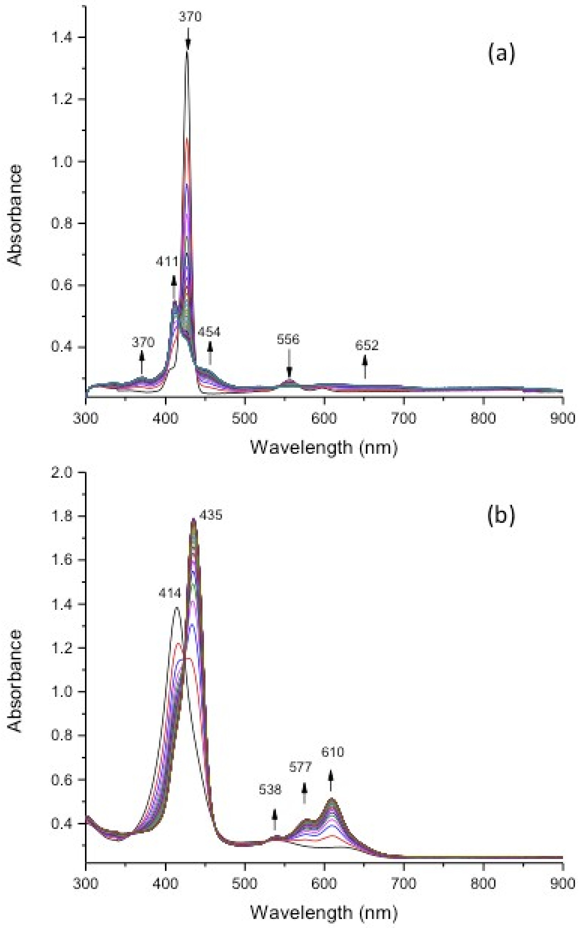

Figure S21: Spectral changes observed during (a) first oxidation of ZnP and (b) CuC in benzonitrile, 0.2 M (TBA)ClO_4.

Femtosecond transient spectral features of the control compounds, ZnP and CuC have been discussed previously[1] and now shown in Figure S**22**. Briefly, excitation of ZnP revealed peaks corresponding to S_0-S_n transitions with peak maxima at 458, 581, 625, and 1280 nm, and minima at 560, 600 and 663 nm due to ground state depletion. The latter two negative peaks also had contributions from stimulated emission. Over a period of time, the singlet peaks decayed in intensity with the appearance of new transient peaks at 474 and 850 nm corresponding to ^{3}ZnP* formed via intersystem crossing. The 1280 nm corresponding to the singlet-singlet transition of ^{1}ZnP* decayed at a rate of 6.03 x 10^8 s^{-1} yielding a time constant of 1.7 ns which in agreement with the lifetime of ZnP in benzonitrile. The nanosecond transient absorption spectra of ^{3}ZnP* further revealed the presence of these peaks with a lifetime of about 77 μs. The transient spectra of CuC revealed spectral features of instantaneously formed but short lived singlet excited CuC with peak maxima at 667 and 609 and 753 nm. In agreement with the non-detectable fluorescence, the transient 609 nm peak decayed at a rate greater than 10^{10} s^{-1} indicating fast intersystem crossing to populate the ^{3}CuC*. The ^{3}CuC* state had peaks at 450 and 670 nm. A decay rate constant of 7.0 x 10^9 s^{-1} for ^{3}CuC* was recorded indicating rapid relaxation of the triplet excited state to the ground state. In summary, both singlet and triplet excited states of CuC were short lived in benzonitrile.

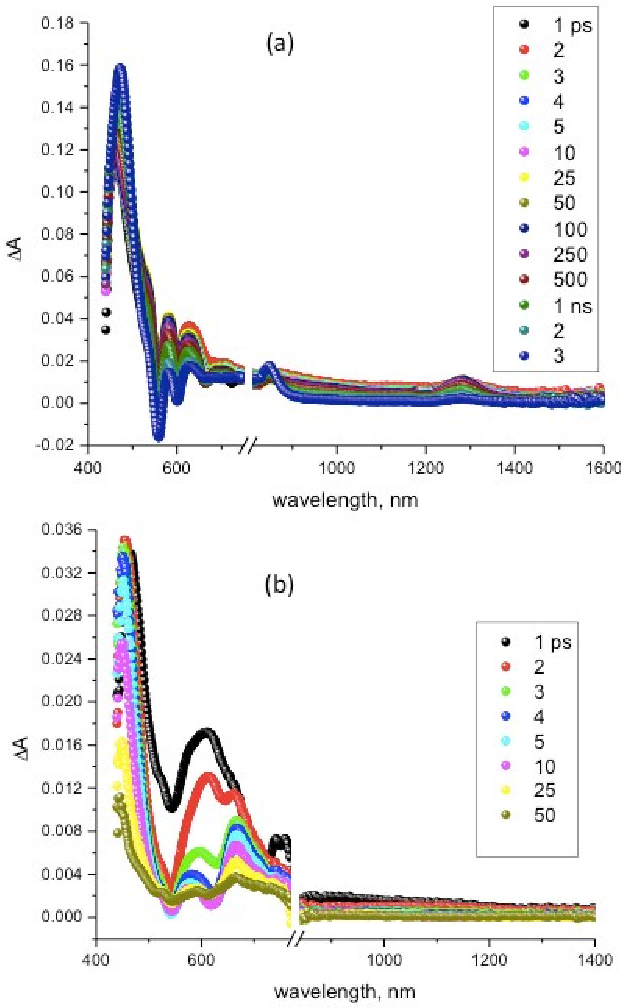

Figure S22: Femtosecond transient absorption spectra at the indicated delay times of (a) ZnP and (b) CuC in benzonitrile (λ_{ex} = 400 nm of 100 fs pulses).

The femtosecond transient spectra of **4⊂3** revealed charge separation in benzonitrile as shown in Figure S23a. Due to the occurrence of the competitive charge separation process, the instantaneously formed ^{1}ZnP* decayed without populating the ^{3}ZnP* triplet state. New transient peaks in the 615 nm region corresponding to Cu^{II}C, and a broad peak in the 625-700 nm region corresponding to the formation of ZnP$^{\bullet+}$ were clearly observed, providing evidence for charge separation in the **4⊂3**. At higher delay times, the intensity of the peaks corresponding to the ZnP$^{\bullet+}$ and Cu^{II}C species diminished suggesting occurrence of charge recombination process.

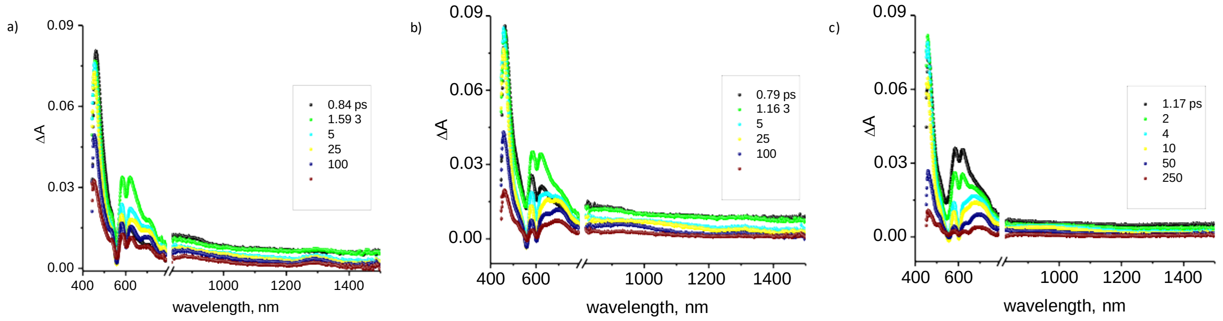

Figure S23: Femtosecond transient absorption spectra of a) **4⊂3**, b) **5⊂3**$_2$ and c) **6⊂3**$_4$ at the indicated delay times (λ_{ex} = 400 nm) in benzonitrile.

The transient spectral features for **5⊂3**$_2$ and **6⊂3**$_4$ were similar to that of **4⊂3** as shown in Figure S23b and c respectively. That is, charge separation from the instantaneously formed

^{1}ZnP* to yield ZnP$^{\bullet+}$-Cu^{II}C within **5⊂3**$_2$ and **6⊂3**$_4$ were observed. In addition, transient peak in the 690 nm range corresponding to ^{3}CuC* was also observed. This has been attributed to ^{3}CuC* formed by direct excitation of CuC to ^{1}CuC* undergoing intersystem crossing to populate ^{3}CuC*.

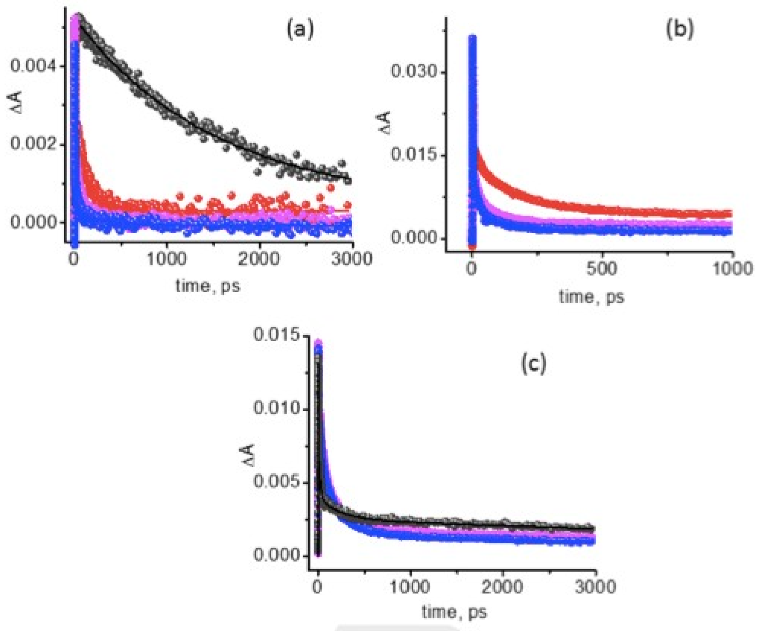

Figure S24: Time profiles of the (a) 1290 nm, (b) 620 nm and (c) 690 nm transient signals of ZnP (black), **4⊂3** (red) **5⊂3**$_2$ (magenta) and **6⊂3**$_4$ (blue).

In order to estimate the kinetics of charge separation and recombination, and to seek the influence of multiple CuC rotors on the charge separation processes, the time profiles of the different signals were systematically analyzed. Figure S24a shows time profiles of the 1290 nm peak representing singlet-singlet excitation of ^{1}ZnP* of control compound ZnP along with the investigated compounds. While the decay of the signal corresponding to ^{1}ZnP* lasted over 3 ns, the monitoring time window of our setup, the corresponding signal of ^{1}ZnP* in the **4⊂3**, **5⊂3**$_2$ and **6⊂3**$_4$ revealed faster decay as a result of the occurrence of photoinduced charge separation process. Such rapid decay followed the trend: **6⊂3**$_4$ < **5⊂3**$_2$ < **4⊂3** < ^{1}ZnP, that is an increased rate of decay with increasing numbers of CuC rotors. Figure S24b shows the time profile of the 620 nm transient peak correponding to Cu^{II}C. Here, rise of this signal provides time constant for charge separation and decay of the signal provides time constants for charge recombination. In all cases, the signal rise was about 1 ps close to the spectrometer detection limit, resulting into k_{CS} values ~10^{11} s^{-1}. The decay of this signal was also fast and followed the number of CuC rotor entities, viz., higher the CuC rotors faster was the decay. The measured time constants **4⊂3**, **5⊂3**$_2$ and **6⊂3**$_4$ were found to be 48.2, 29.3 and 25.5 ps respectively. This yielded k_{CR} values of 2.1 x 10^{10} s^{-1} and 3.4 x 10^9 s^{-1}, and 3.9 x 10^9 s^{-1}, respectively, for **4⊂3**, **5⊂3**$_2$ and **6⊂3**$_4$. Finally, the decay of the 690 nm peak corresponding to ^{3}CuC* was also monitored as shown in Figure S24c to evaluate k_T values. The k_T values were 3.8 x 10^9 s^{-1} for **5⊂3**$_2$, and 6.0 x 10^9 s^{-1} for **6⊂3**$_4$ close to that of pristine CuC (7.0 x 10^9 s^{-1}).

The photochemical behaviors of **4⊂3**, **5⊂3**$_2$ and **6⊂3**$_4$ described above directly parallel those of non-interlocked axles **4**, **5** and **6**[1] and support the conclusion that formation of the mechanical bond does not significantly perturb the electronic structure of these systems.

5. Space Filling Models of $4\subset3$, $5\subset3_2$,$6\subset3_4$, $[(6\subset3_4)L]$ and $[(6\subset3_4)_2L]$

Models of [2]rotaxane **4⊂3**, [3]rotaxane **5⊂3**$_2$ and [5]rotaxane **6⊂3**$_4$ (Figures S25-27 respectively) were generated using Spartan '10 (Wavefunction Inc.), minimized using MMFF (the axle was constructed in a highly symmetrical planar conformer and the macrocycle located over the triazole prior to optimization) and compared to the corresponding non-interlocked axles. These models clearly show the sterically hindered environment around their Zn porphyrin cores.

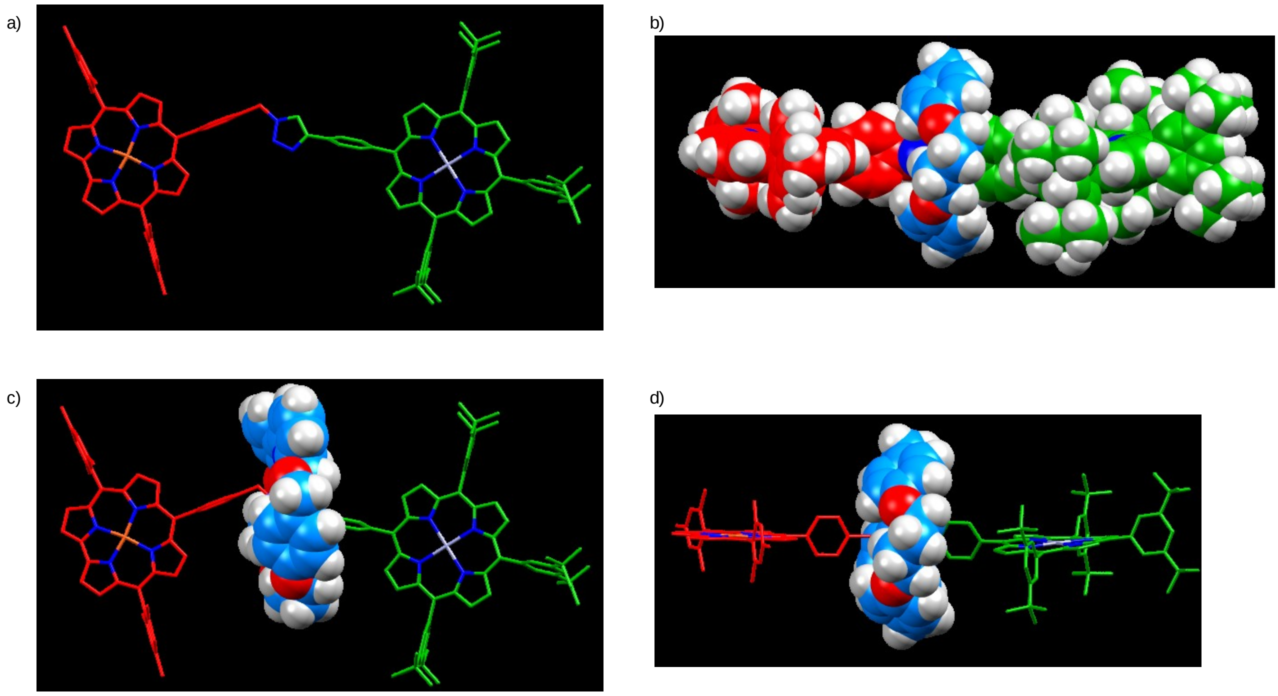

Figure S25. Comparison of a) axle **4** (sticks representation) and [2]rotaxane **4⊂3** (various views b-d).

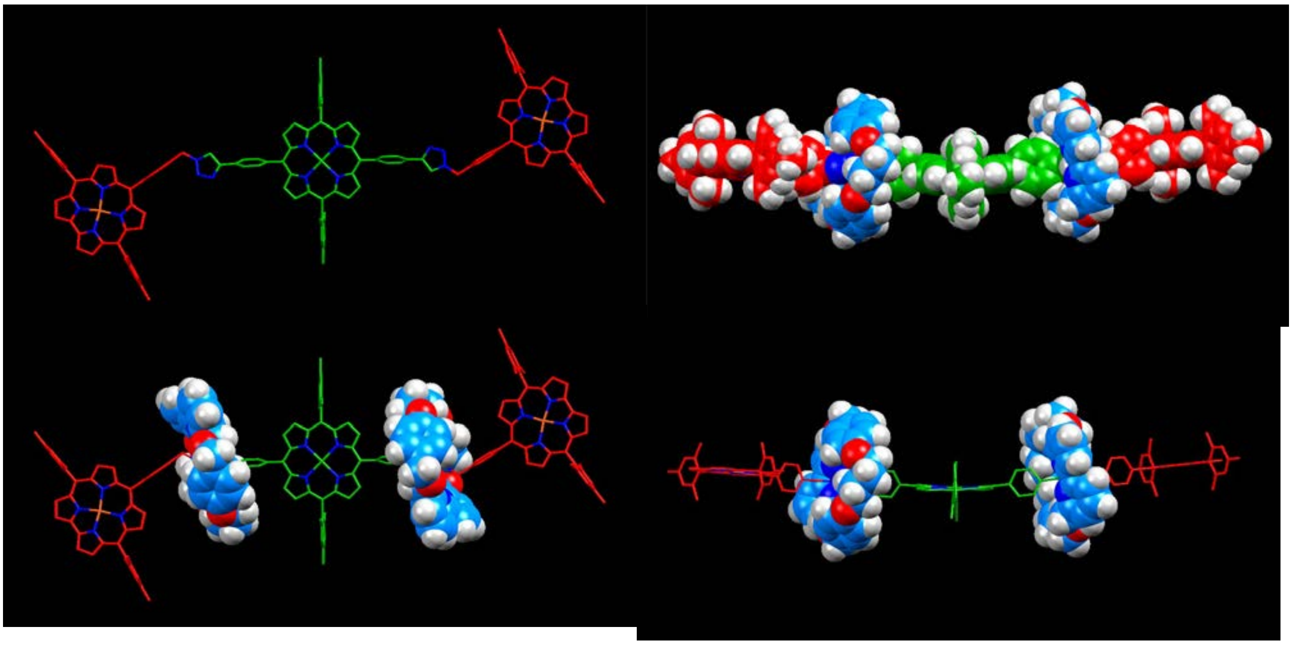

Figure S26. Comparison of a) axle **5** (sticks representation) and [3]rotaxane **5⊂3**$_2$ (various views b-d).

a) b

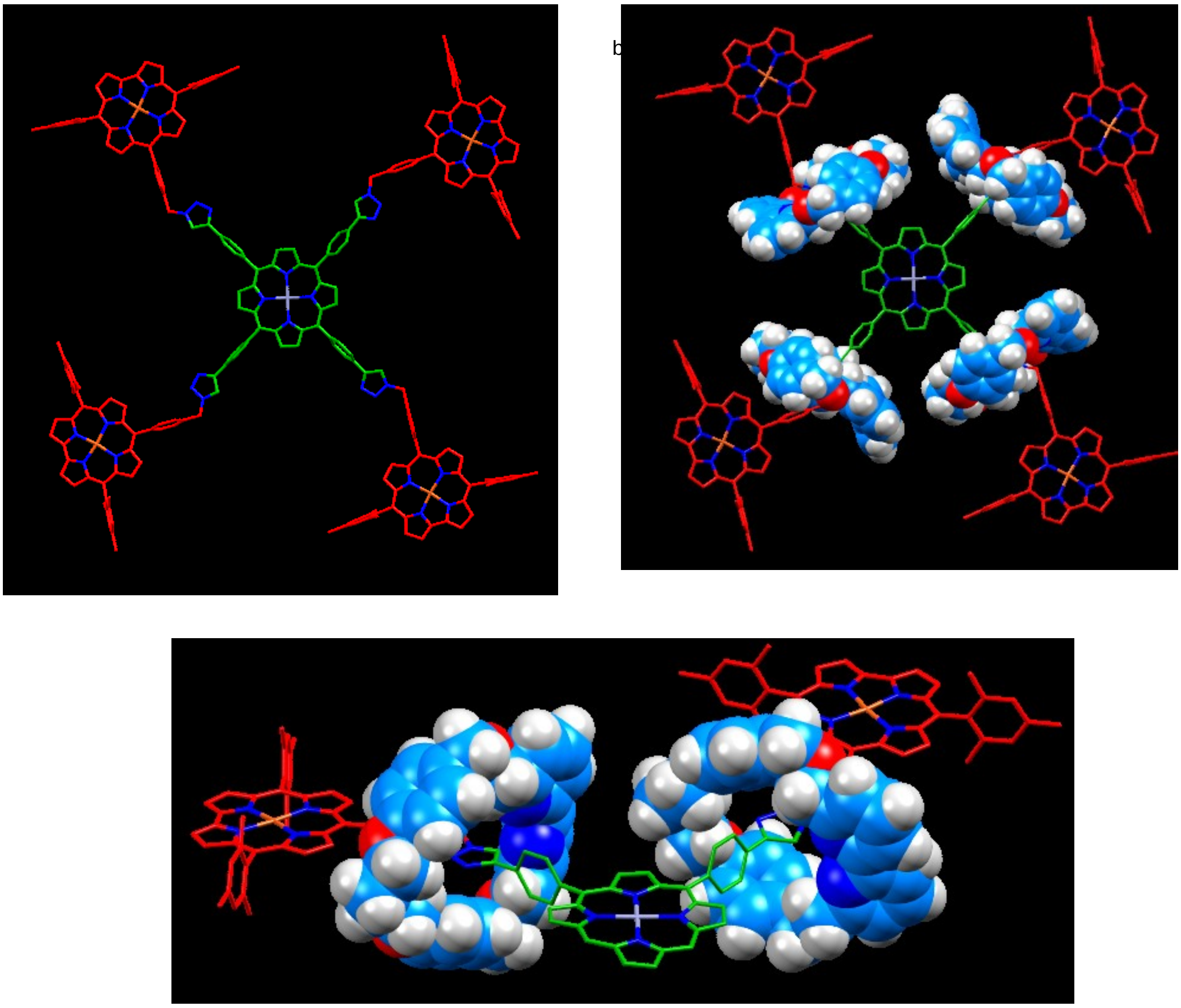

Figure S27. Comparison of a) axle **6** (sticks representation) and b) [5]rotaxane $\mathbf{6}\subset\mathbf{3}_4$ (axle in sticks representation, macrocycle in spacefill; top view).c) truncated model of rotaxane $\mathbf{6}\subset\mathbf{3}_4$ with two “arms” removed showing crowding of porphyrin unit by macrocycles.

The model of $\mathbf{6}\subset\mathbf{3}_4$ shown in Figure S27 was used to generate models of moneric complex [($\mathbf{6}\subset\mathbf{3}_4$)L] and dimeric complex [($\mathbf{6}\subset\mathbf{3}_4$)$_2$L]. As can be seen, DABCO occupies a relatively unhindered region in the monomeric complex as the macrocycles are positioned approximately 3.5 Å away (based on %Vbur calculations – see below). However, formation of [($\mathbf{6}\subset\mathbf{3}_4$)$_2$L imposes significant steric interactions leading to a rotation of the porphyrin units relative to one another and significant steric clash between the two units of $\mathbf{6}\subset\mathbf{3}_4$ (Figure S28).

a) b)

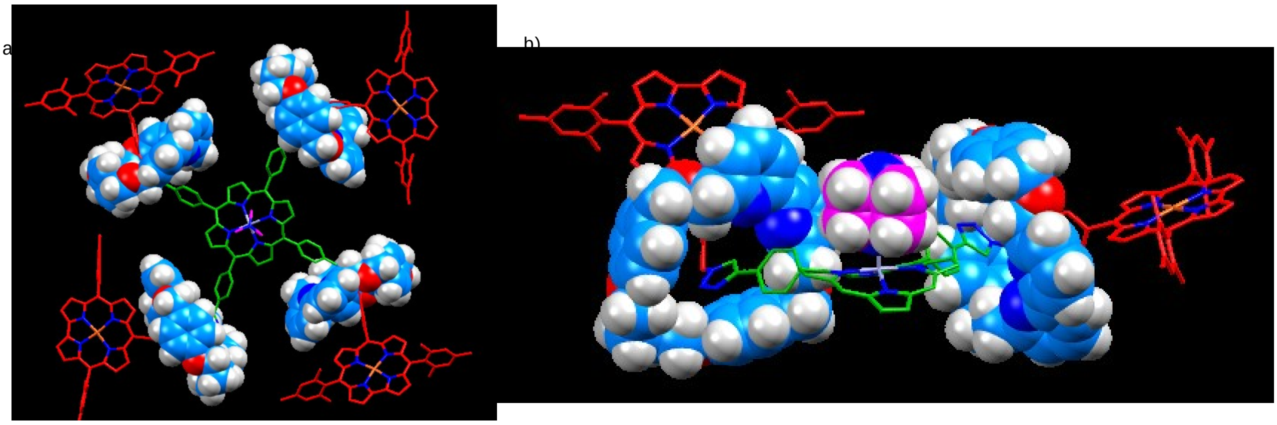

Figure S28. Monomeric complex $[(6\subset 3_4)L]$ viewed from the top (a; axle and DABCO unit in sticks representation, macrocycle in spacefill) and side (b); truncated to remove 2 “arms”, axle in sticks representation, macrocycle and DABCO unit in spacefill).

c)

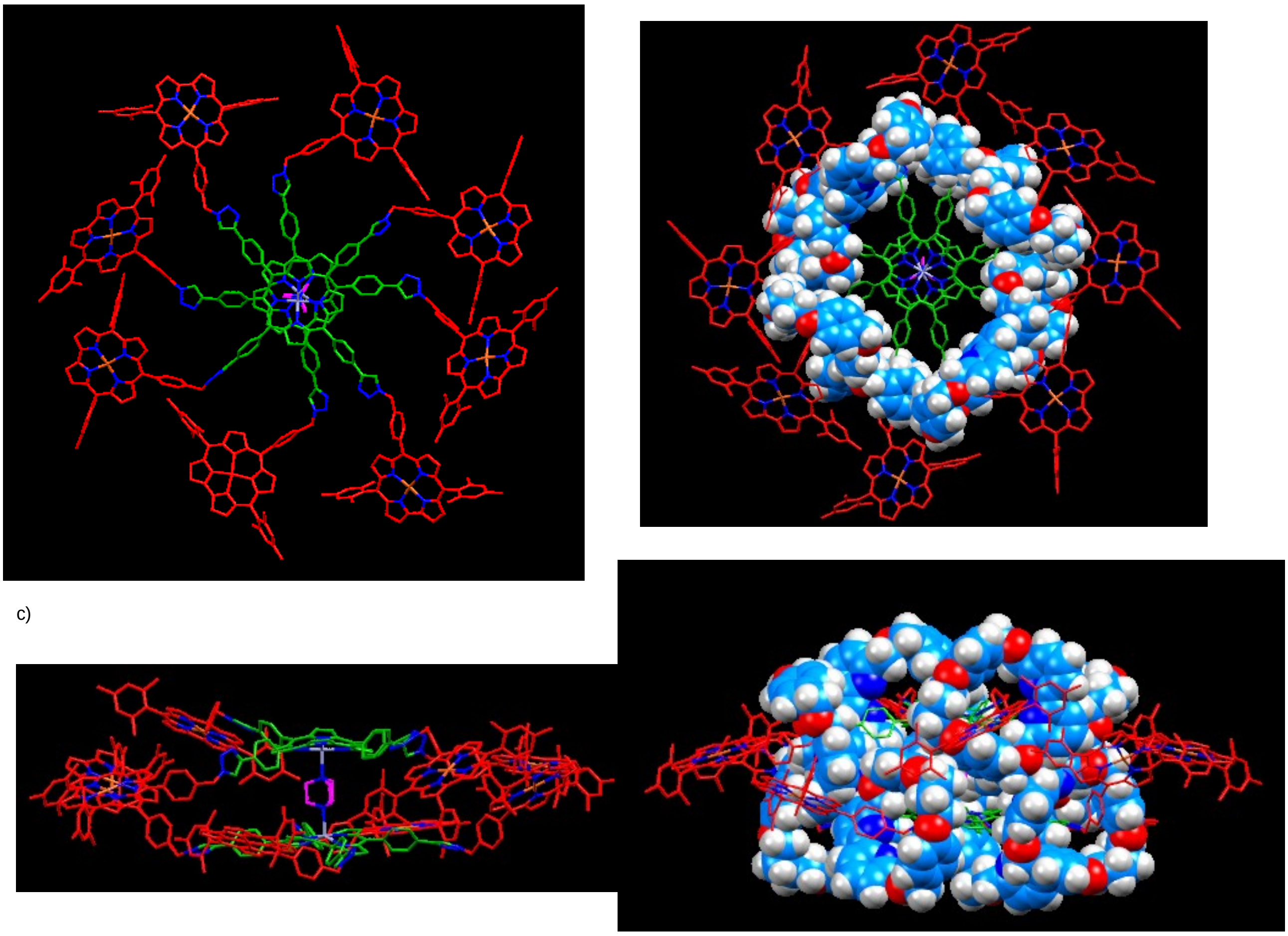

Figure S29. Dimeric complex $[(\mathbf{6})_2L]$ in sticks representation viewed from the a) top and b) side and dimeric complex $[(\mathbf{6}\subset\mathbf{3}_4)_2L]$ with axle component in sticks representation and macrocycle in spacefill viewed from the b) top and c) side.

6. % Buried Volume (%Vbur) of Axle 6 and Rotaxane $\mathbf{6{\subset}3_4}$

Truncated models of axle **6** and rotaxane $\mathbf{6{\subset}3_4}$ (Figure S25) with the corrole units removed were generated using Spartan ’10 (Wavefunction Inc.). The models were then edited to replace Zn with H (as required for %Vbur calculations) and the values of %Vbur for spheres centered on the Zn coordinate were calculated for a range of radii using the SambVca web application developed by Cavallo and co-workers.[6]

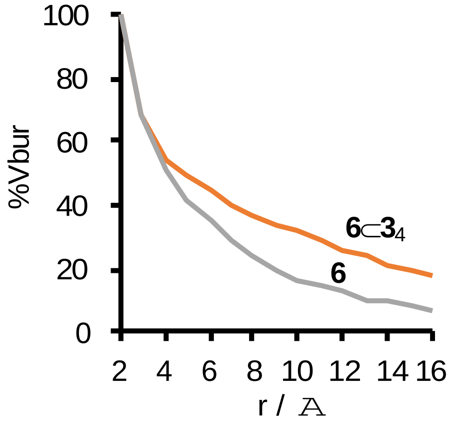

Figure S30: Variation of variation of %Vbur of a sphere of radius r centered on Zn with respect to r.

Table S1: values of buried volume and % buried volume (%Vbur) for truncated models of axle **6** and rotaxane $\mathbf{6{\subset}3_4}$.

		Axle **6**		Rotaxane $\mathbf{6{\subset}3_4}$	
r (Å)	total V (Å^3)	buried V (Å^3)	%Vbur	buried V (Å^3)	%Vbur
2	33.469	33.346	99.63249574	33.346	99.51059385
2.5	65.406	53.625	81.98789102	53.625	81.98789102
3	113.007	77.223	68.33470493	77.187	68.3028485
3.5	179.535	105.943	59.00966385	104.025	57.92231366
4	267.954	144.274	53.84282377	136.079	50.784463
4.5	381.678	194.303	50.9075713	175.579	46.00186545
5	523.475	254.904	48.69458904	220.441	42.11108458
6	904.568	398.264	44.02808855	318.993	35.26467883
7	1436.497	570.868	39.74028487	419.179	29.18063873
8	2144.414	789.408	36.81229464	514.92	24.01215437
9	3053.39	1045.958	34.25563063	609.563	19.96348321
10	4188.478	1333.84	31.84545794	707.782	16.89831008
11	5574.859	1599.013	28.68257296	803.576	14.41428384
12	7237.812	1845.959	25.50437895	896.788	12.39031906
13	9202.359	2153.776	23.40460745	983.511	10.68759652
14	11493.461	2496.711	21.72288225	1064.531	9.262057791
15	14136.775	2820.442	19.95109917	1128.257	7.981006984
16	17156.763	3123.882	18.20787523	1177.439	6.862827213

7. Variable Concentration and Temperature ^{1}H NMR of Axle 6 and [5]rotaxane 6⊂3$_4$

As the concentration increases, broadening of most chemical shift of signals of thread **6** was observed (**Figure S 31**), indicating the intermolecular π-π stacking occurs between the porphyrin cores and corrole periphery. Such aggregation is prevented by the bulky macrocycles in rotaxane **6⊂3**$_4$ (**Figure S 32**), hence the signal here remain sharp with the increasing concentration.

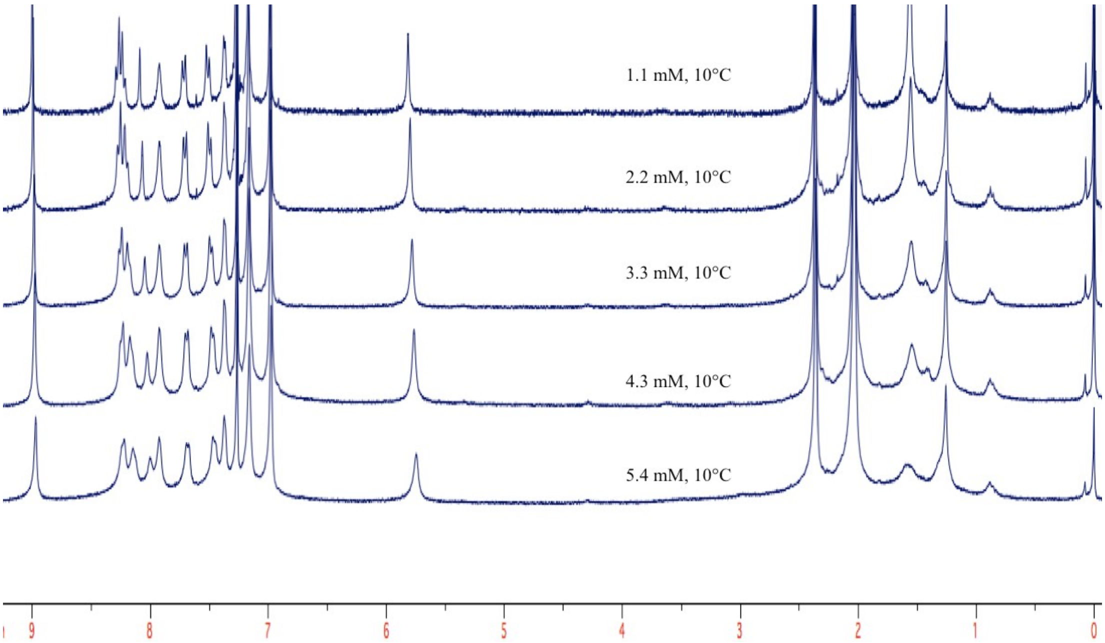

Figure S 31: Concentration dependent chemical shifts of thread **6** at 10°C.

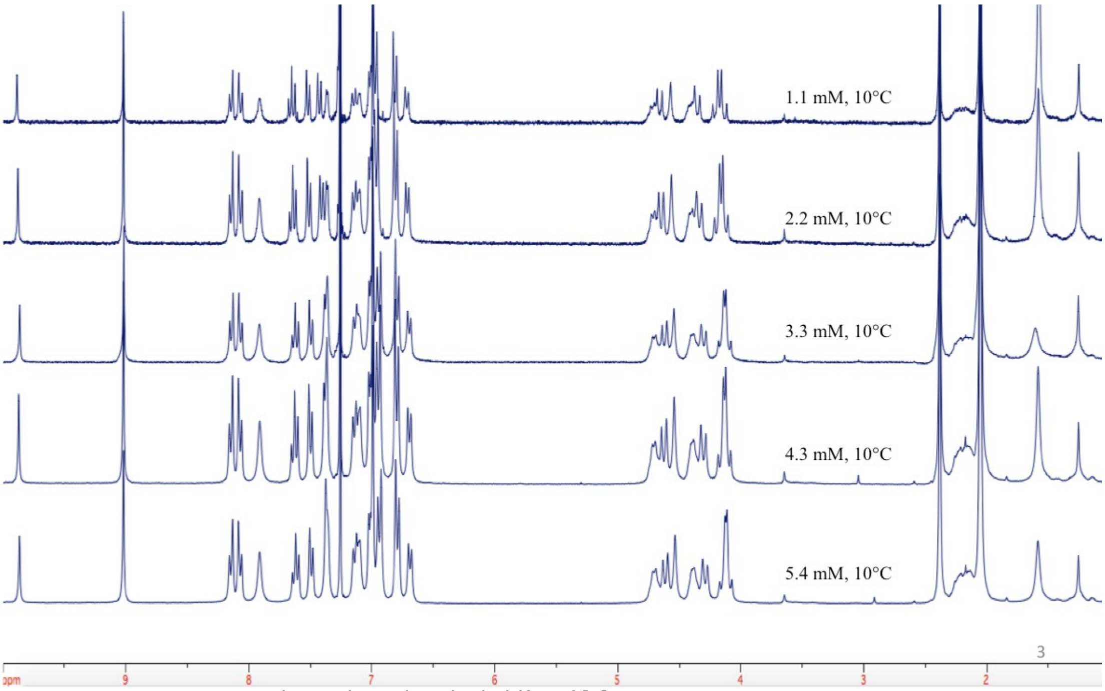

Figure S 32: Concentration dependent chemical shifts of [5]rotaxane **6⊂3**$_4$ at 10°C.

Variable temperature ^{1}H NMR spectroscopy was performed for [5]rotaxane **6⊂3**$_4$. The chemical shift of signals of rotaxane **6⊂3**$_4$ remained consistent (with the exception of H_k and H_i which are presumably influenced by macrocycle mobility which is affected by temperature) as the temperature was varied, although they became broadened at low temperature, presumably due to reduced molecular tumbling.

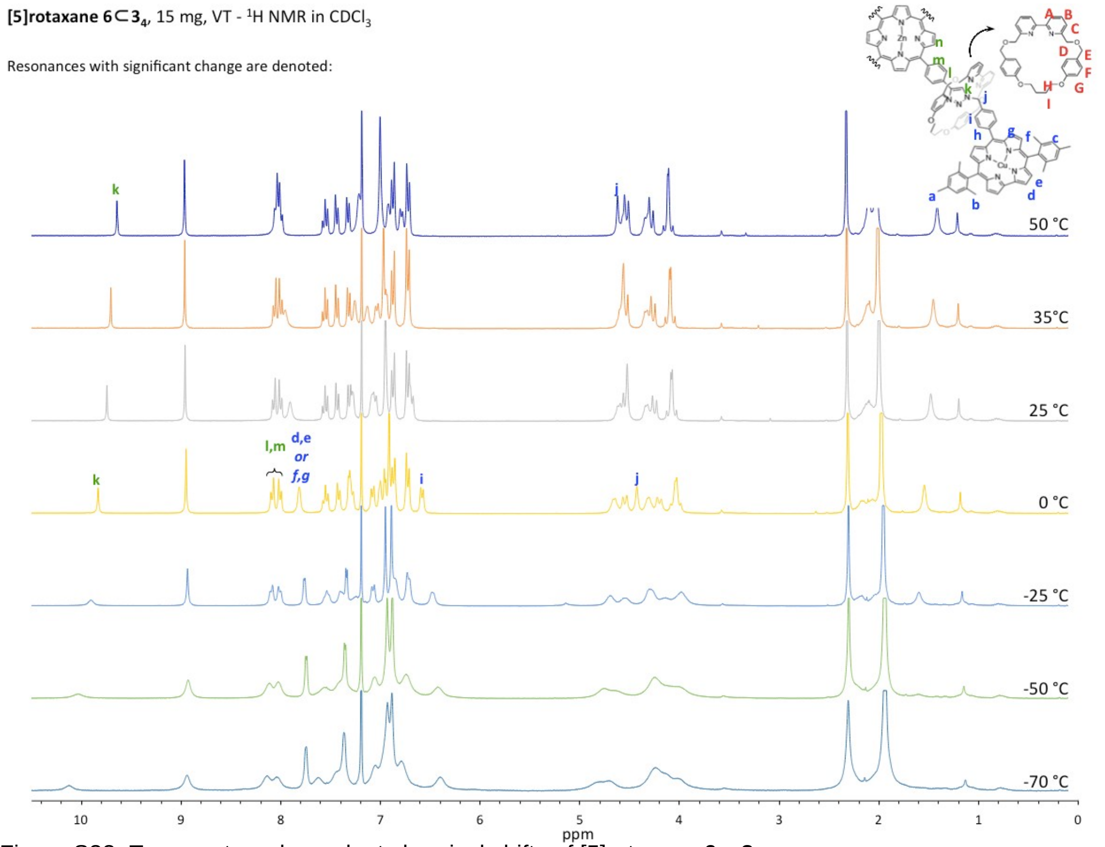

Figure S33: Temperature dependent chemical shifts of [5]rotaxane **6⊂3**$_4$

8. DABCO Titrations

Rotaxane $6\subset3_4$ titration with DABCO (L)

The binding properties of **$6\subset3_4$** and DABCO were investigated using ^{1}H NMR titration experiment (Figure S**34**). Relative chemical shift of two resonances (denoted as k and n in Figure S**34**) $\Delta\delta_{obs} = \delta_k - \delta_n$ is observed. The models below were interpreted as indicating binding of DABCO to the Zn porphyrin centre rather than the Cu corrole moieties. This is justified as a) the binding stoichiometry suggests a 1 : 1 interaction at high ratios of L : Zn (as opposed to a > 4 : 1 stoichiometry that would be expected if the Cu-corrole interacted significantly with the corrole moieties) and b) a literature search revealed no previous examples of exogenous ligands binding to Cu-corrole moieties.

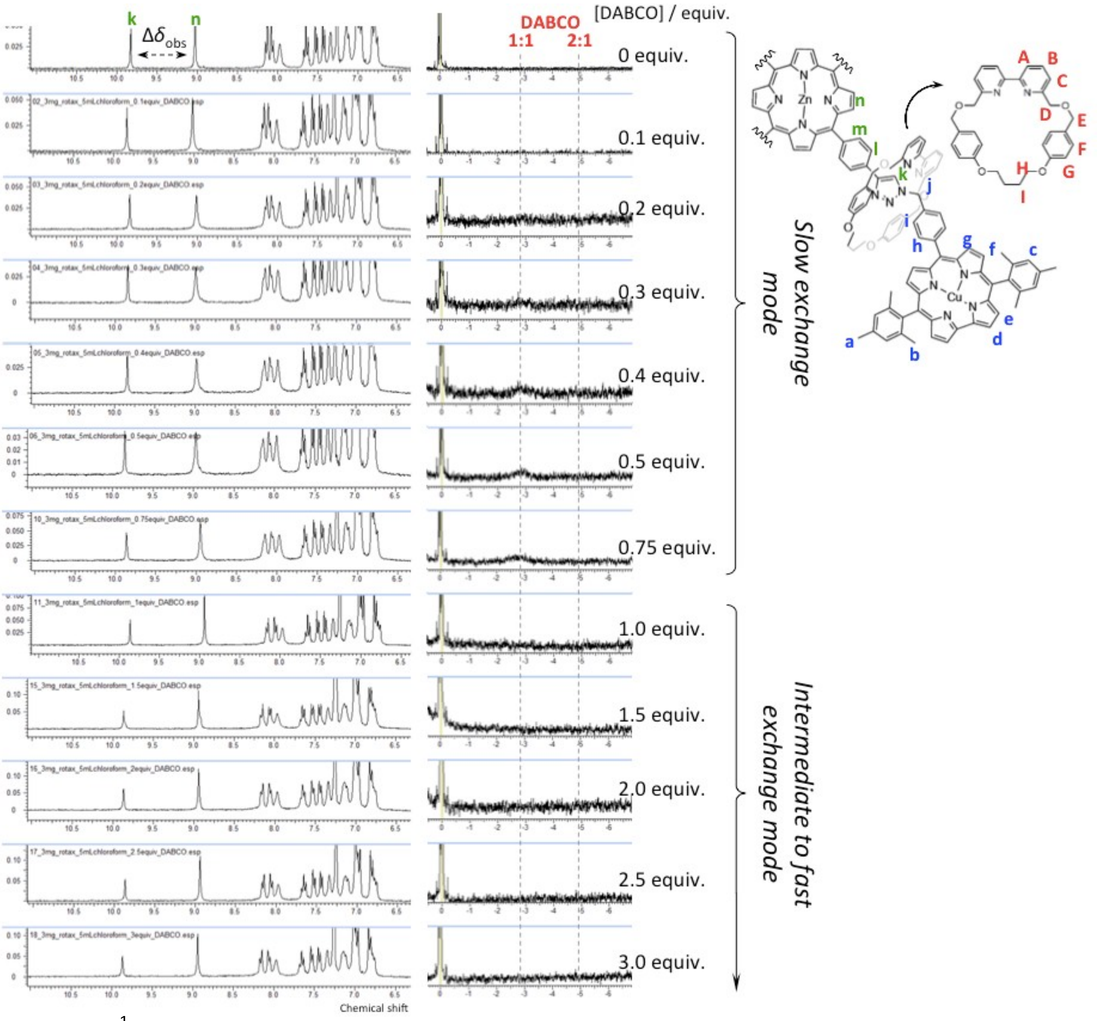

Figure S34: ^{1}H NMR titration experiment of host rotaxane **$6\subset3_4$** (1.1 mM, chloroform, 25 °C) with guest DABCO (L). Dashed lines indicate positions of DABCO resonances corresponding to 1:1 [**$(6\subset3_4)$L**] and 2:1 (not present) [**$(6\subset3_4)_2$L**] complex forms.

1:1 **[$(6\subset3_4)$L]** model was adopted (see Scheme S**1** for equilibrium equation).

$$(6\subset3_4) + \mathrm{L} \underset{slow/fast}{\overset{K_1}{\rightleftarrows}} [(6\subset3_4)\mathrm{L}]$$

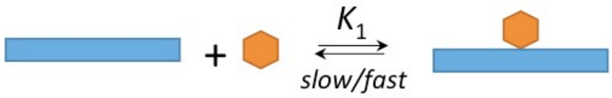

Scheme S1: Equilibrium equation of Rotaxane (**$6\subset3_4$,** rectangle) and DABCO (**L**, hexagon).

Where K_1 is equilibrium binding constant of formation of **[(6⊂3₄)L]** complex. Equilibrium binding constant, mass balance equations and binding isotherm are then expressed in eqns. E11-E15.

$$K_1 = \frac{[(6 \subset 3_4)L]}{[6 \subset 3_4][L]} \quad \text{(E11)}$$

$$[L]_t = [L] + [(6 \subset 3_4)L] \quad \text{(E12)}$$

$$[6 \subset 3_4]_t = [6 \subset 3_4] + [(6 \subset 3_4)] \quad \text{(E13)}$$

$$K_1[6 \subset 3_4]^2 + K_1\left(1 + [L]_t - [6 \subset 3_4]_t\right)[6 \subset 3_4] - [6 \subset 3_4]_t = 0 \quad \text{(E14)}$$

$$\Delta\delta_{obs} = \Delta\delta_{(6 \subset 3\_4)L}[(6 \subset 3_4)L]/[6 \subset 3_4]_t \quad \text{(E15)}$$

Where $[\mathbf{6 \subset 3_4}]_t$ and $[L]_t$ denote total concentration of $\mathbf{6 \subset 3_4}$ and **L**, respectively. $[\mathbf{6 \subset 3_4}]$ and [**L**] are concentration of free $\mathbf{6 \subset 3_4}$ and **L**, respectively. $[\mathbf{(6 \subset 3_4)L}]$ denotes concentration of complexed $\mathbf{(6 \subset 3_4)L}$ form. $\Delta\delta_{obs}$ and $\Delta\delta_{(6 \subset 3\_4)L}$ are observed chemical shift difference and chemical shift difference between $\mathbf{(6 \subset 3_4)L}$ complexed and $\mathbf{6 \subset 3_4}$ free form, respectively.

The model presented fits well to observed data (Figure S**35**a) with lower boundary for $K_1 \geq 2 \times 10^5$ M^{-1} ($\Delta\delta_{(6 \subset 3\_4)L}$ = 0.126 ppm). The binding constant is relatively high and its exact value cannot be determined using NMR titration technique. Note that in spectra at 25 °C (Figure S**34**) there is no indication of 2:1 $\mathbf{[(6 \subset 3_4)_2L]}$ complex (contrary to Thread/DABCO system) and moreover Figure S**35**a clearly indicates 1:1 $\mathbf{[(6 \subset 3_4)L]}$ stoichiometry.

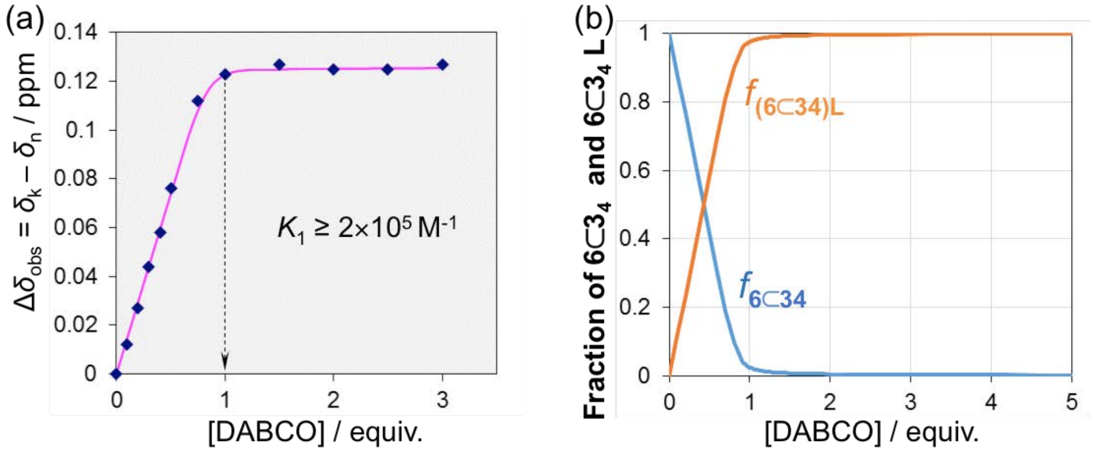

Figure S35: (a) Plot of experimentally observed values of chemical shift difference $\Delta\delta_{obs}$ (blue diamonds) as obtained from titration experiment (Figure S34). Magenta line represents fit of eqns. E11-E15 (K_1 = 2×10^5 M^{-1}, $\Delta\delta_{(6 \subset 3\_4)L}$ = 0.126 ppm). (b) Calculated fractions of free ($f_{6 \subset 3\_4}$) and complexed ($f_{(6 \subset 3\_4)L}$) Rotaxane as a function of DABCO concentration. Eqns. E11-E15 are used with lower limit of K_1 = 2×10^5 M^{-1}.

Non-interlocked pentad 6 titration with DABCO (L)

The binding properties of host (**6**) thread and guest (**L**) DABCO were investigated using ^{1}H NMR titration experiment (Figure S**36**). Two quantities are observed. Fraction of both complexed forms (1:1 **[6L]** and 2:1 $\mathbf{[6_2L]}$) of host **6** $f_{obs} = I_{n'}/(I_n + I_{n'})$. Where I_n and $I_{n'}$ are integrated intensities of *beta*-protons corresponding to free and (both) complexed forms of host **6**, respectively (see Figure S**36** for n and n' resonances). Also absolute chemical shift $\delta_{obs,n'}$ of n' resonance is recorded.

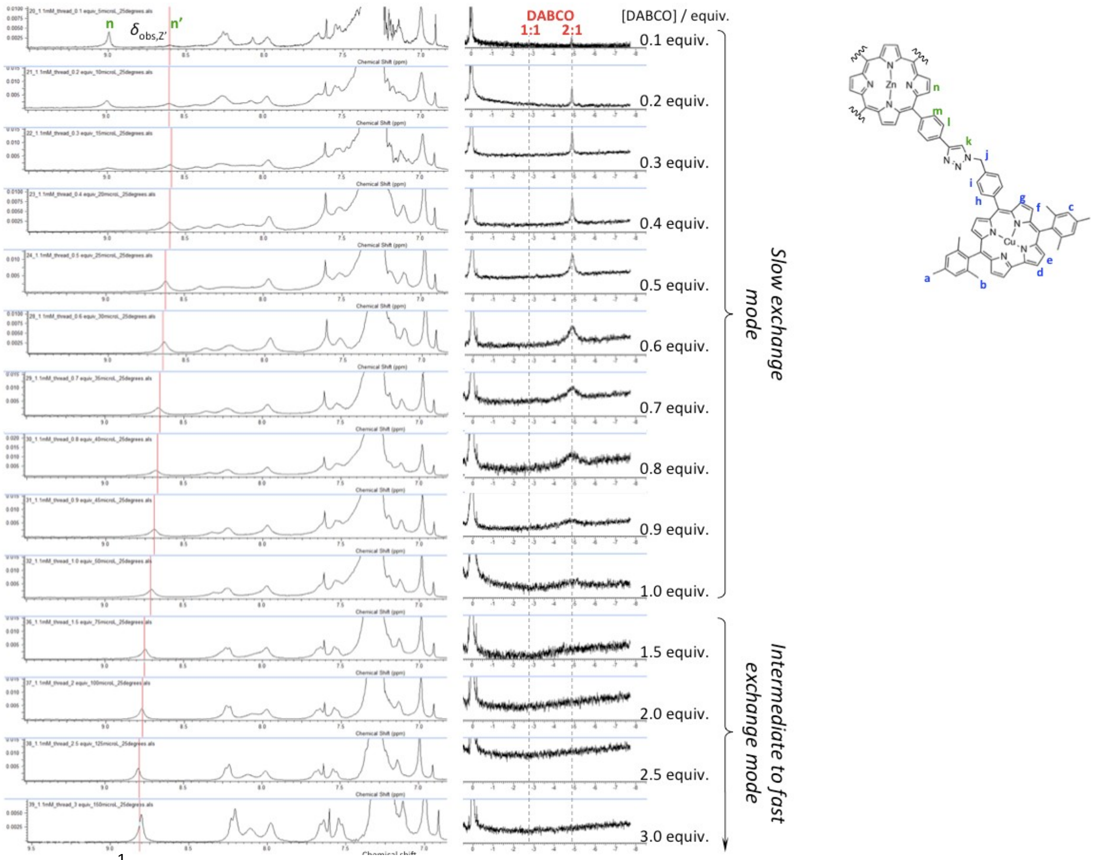

Figure S36: ^{1}H NMR titration experiment of host **6** (1.1 mM, chloroform, 25 °C) with guest ligand (**L**) DABCO. Dashed lines indicate positions of DABCO resonances corresponding to 1:1 [**6L**] (not present at 25 °C) and 2:1 [**6$_2$L**] complex forms.

2:1 **[6$_2$L]** model was adopted (see Scheme S**2** equilibrium equation).

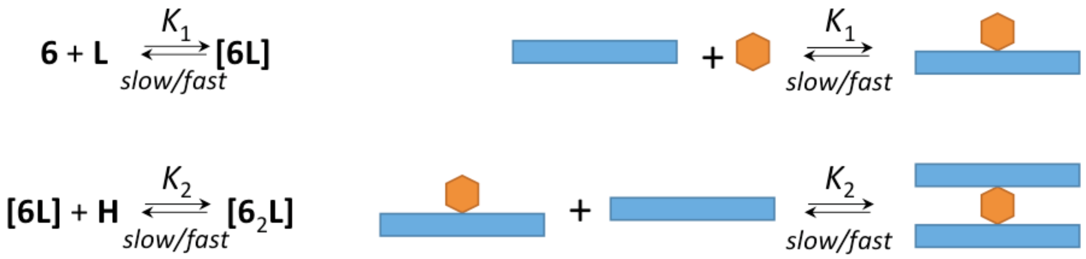

Scheme S2: Equilibrium equations of thread (**6**, rectangle) and DABCO (**L**, hexagon).

Where K_1 and K_2 are equilibrium binding constants of formation of **[6L]** and **[6$_2$L]** complexes, respectively. Equilibrium binding constants, mass balance equations and binding isotherms are then expressed in eqns. E16-E22. This treatment is similar to that in Ref. [7].

$$K_1 = \frac{[6L]}{[6][L]} \quad \text{(E16)}$$

$$K_2 = \frac{[6_2L]}{[6L][L]} \quad \text{(E17)}$$

$$[L]_t = [L] + [6L] + [6_2L] \quad \text{(E18)}$$

$$[6]_t = [6] + [6L] + 2[6_2L] \quad \text{(E19)}$$

$$K_1 K_2 [6]^3 + K_1\left(1 + 2K_2[L]_t - K_2[6]_t\right)[6]^2 + \left(1 + K_1[L]_t - K_1[6]_t\right)[6] - [6]_t = 0 \quad \text{(E20)}$$

$$f_{obs} = \left([6L] + 2[6_2L]\right)/[6]_t \quad \text{(E21)}$$

$$\delta_{obs,n'} = \left(\delta_{6L}[6L] + 2\delta_{62L}[6_2L]\right)/\left([6L] + 2[6_2L]\right) \quad \text{(E22)}$$

Where [**6**]$_t$, [**L**]$_t$, [**6**], [**L**] and [**6L**] has its usual meaning (see above) and [**6$_2$L**] denotes concentration of ternary complex **6$_2$L**. δ_{6L} and δ_{6_2L} are chemical shifts of **[6L]** and **[6$_2$L]** complex, respectively.

The model was simultaneously fitted to both binding isotherms (f_{obs} and $\delta_{obs,n'}$) as seen in Figure S37a,b. Only lower boundary of binding constants (not its exact value) can be obtained from NMR titration (unlike in Ref. [7], where additional UV/Vis titration experiments were used). The lower boundary for K_1 is $K_1 \geq 5\times10^6$ M^{-1} and K_2 dependence on K_1 can be estimated as $K_2 = 5.27\times K_1^{0.98} \approx 3.8\ K_1$ (δ_{6L} = 9.08 ppm, δ_{6_2L} = 8.60 ppm). This yields positive cooperativity $\alpha = 4K_2/K_1 \approx 15$. The lower boundary of K_1 is about an order of magnitude higher for **6**/DABCO system compared to Rotaxane/DABCO at 25 °C. Again, note that in spectra at 25 °C (Figure S36) there is no indication of 1:1 **[6L]** complex (contrary to Rotaxane/DABCO system) and moreover Figure S37a,b clearly indicates only 2:1 **[6L]** stoichiometry. At low DABCO concentration the exchange of DABCO at **6**'s binding place is slow. It became intermediate/fast at round 1 equiv. of DABCO. Therefore, formation of 1:1 complex cannot be seen around -3 ppm due to intermediate/fast exchange at higher DABCO concentration. At high DABCO concentration (despite the positive cooperativity α≈15) the 1:1 **[6L]** complex becomes dominant.

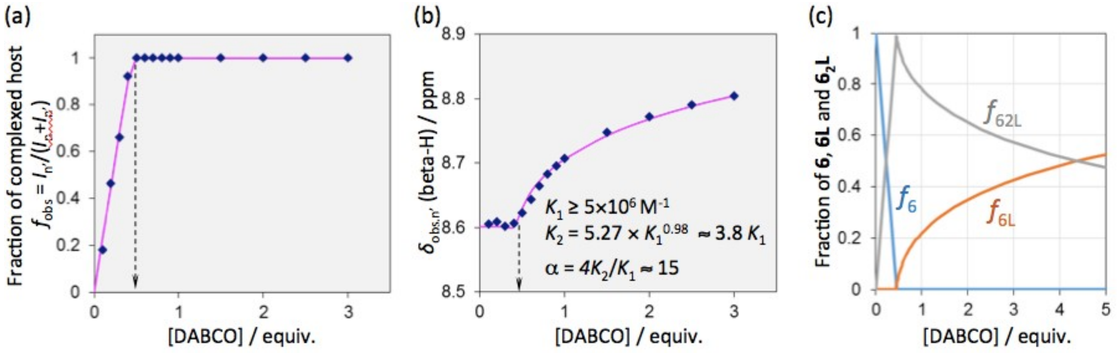

Figure S37: (a) Plot of experimentally observed values of fraction of both complexed forms of host f_{obs} (blue diamonds) as obtained from titration experiment (Figure S36) (b) Experimentally observed chemical shift $\delta_{obs,n'}$ of host's *beta*-protons (blue diamonds). Magenta lines represents fit of eqns. E16-E22 ($K_1 \geq 5\times10^6$ M^{-1}, $K_2 = 5.27\times K_1^{0.98} \approx 3.8\ K_1$, δ_{6L} = 9.08 ppm, δ_{6_2L} = 8.60 ppm, $\alpha = 4K_2/K_1 \approx 15$). (c) Calculated fractions of free (f_6) and complexed (f_{6L} and f_{6_2L}) thread **6** forms as a function of DABCO concentration. Eqns. E16-E22 are used with lower limit of $K_1 = 5\times10^6$ M^{-1} and $K_2 = 2\times10^7$ M^{-1}.

Complexation preferences in mixed Rotaxane/DABCO/Thread system

Qualitative variable temperature (VT) ^{1}H NMR study of mixed Rotaxane (**6⊂3$_4$**)/DABCO(**L**)/Thread(**6**) system (in slow exchange mode, i.e. at low DABCO concentration) shows several preferences of complex formation. Figure **S38** shows VT stacks of ^{1}H NMR spectra for two systems without and with presence of thread (**6**). The rotaxane/DABCO system show formation of both 1:1 **[(6⊂3$_4$)L]** and 2:1 **[(6⊂3$_4$)$_2$L]** complexes at low temperature (at -3 and -4.8 ppm respectively) with increasing ratio of 2:1 **[(6⊂3$_4$)$_2$L]** complex upon cooling (**Figure S38**a). Note that resonance at -3 ppm corresponds to three CH_2 DABCO protons and resonance at -4.8 ppm corresponds to all six CH_2 groups. The mixed rotaxane/DABCO/thread system upon cooling show presence of 1:1 **[(6⊂3$_4$)L]** and 2:1 **[(6⊂3$_4$)$_2$L]** complexes as well as 2:1 **[6$_2$L]** complex (**Figure S38**b). Interestingly, there is no observable resonance corresponding to mixed ternary **[(6⊂3$_4$)$_2$L6]** complex. This is due to DABCO's high affinity for formation of ternary 2:1 **6$_2$L** complex first (high K_1 and K_2) and then forming 1:1 **[(6⊂3$_4$)L]** and 2:1 **[(6⊂3$_4$)$_2$L]** (lower K_1 and K_2) complexes. In other words, DABCO preferentially binds to all available **6** (forming **6$_2$L** complexes) and remaining (if any) DABCO binds to **6⊂3$_4$** (forming both **[(6⊂3$_4$)L]** and **[(6⊂3$_4$)$_2$L]** complexes). This

behaviour can be qualitatively seen in Figure S38b. The thread (**6**) content determines DABCO's placement in particular type of complex. This is well illustrated in Figure S**39** ([(**6⊂3**$_4$):**L**:**6**] = 1:1:1) where ca. 50 % of **L** is in **6**$_2$**L** form and remaining ca. 50 % of **L** is in [(**6⊂3**$_4$)**L**] and [(**6⊂3**$_4$)$_2$**L**] forms.

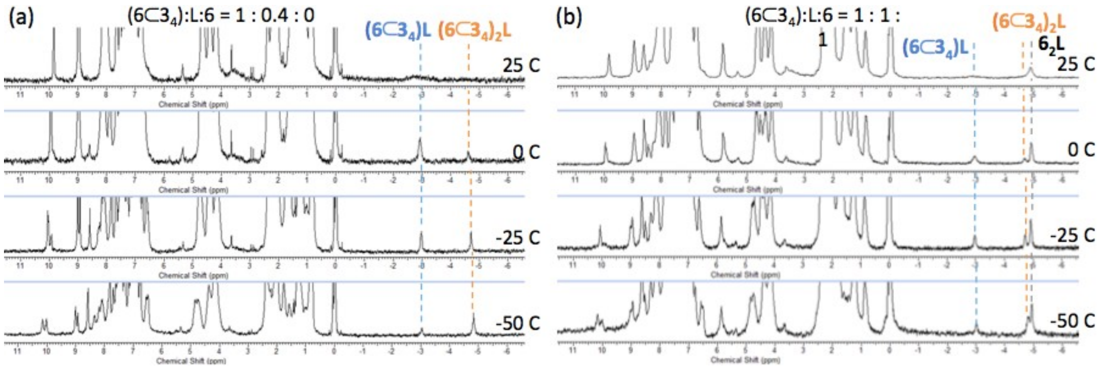

Figure S38: VT ^{1}H NMR spectra of Rotaxane/DABCO/Thread system in chloroform with two compositions (a) [(**6⊂3**$_4$):**L**:**6**] = 1 : 0.4 : 0 and (b) [(**6⊂3**$_4$):**L**:**6**] = 1 : 1 : 1 (for both [**6⊂3**$_4$] = 1.1 mM). Dashed lines indicate positions of DABCO resonances corresponding to [(**6⊂3**$_4$)**L**], [(**6⊂3**$_4$)$_2$**L**] and **6**$_2$**L** complex forms.

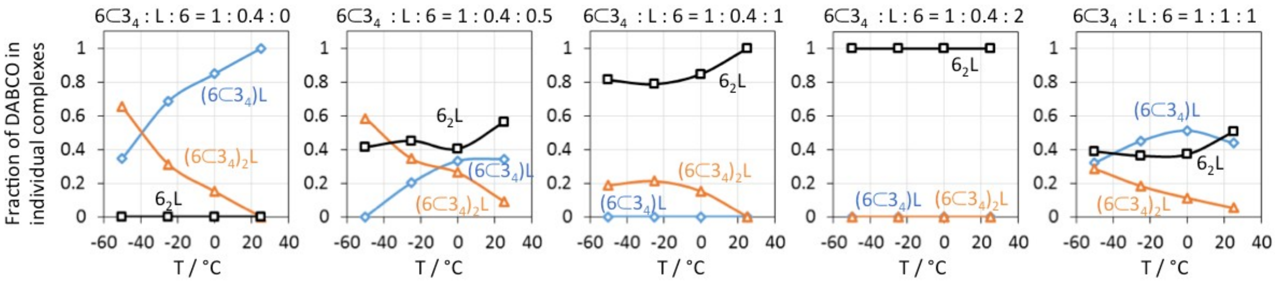

Figure S39: Plots of fraction of DABCO in individual complexes for Rotaxane/DABCO/Thread system with various (**6⊂3**$_4$):**L**:**6** composition (indicated above plots). Data are obtained from integration of resonances corresponding to DABCO in VT ^{1}H NMR experiments (e.g. Figure S38). For all experiments [(**6⊂3**$_4$)] = 1.1 mM.

9. References

1) T. H. Ngo, D. Zieba, W. A. Webre, G. N. Lim, P. A. Karr, S. Kord, S. Jin, K. Ariga, M. Galli and S. Goldup, et al., *Chem. Eur. J.* 2016, **22**, 1301–1312.

2) J. E. M. Lewis, R. J. Bordoli, M. Denis, C. J. Fletcher, M. Galli, E. A. Neal, E. M. Rochette and S. M. Goldup, *Chem. Sci.* 2016, *7*, 3154–3161.

3) Gaussian 09, Revision D.01, M. J. Frisch, G. W. Trucks, H. B. Schlegel, G. E. Scuseria, M. A. Robb, J. R. Cheeseman, G. Scalmani, V. Barone, B. Mennucci, G. A. Petersson, H. Nakatsuji, M. Caricato, X. Li, H. P. Hratchian, A. F. Izmaylov, J. Bloino, G. Zheng, J. L. Sonnenberg, M. Hada, M. Ehara, K. Toyota, R. Fukuda, J. Hasegawa, M. Ishida, T. Nakajima, Y. Honda, O. Kitao, H. Nakai, T. Vreven, J. A. Montgomery, Jr., J. E. Peralta, F. Ogliaro, M. Bearpark, J. J. Heyd, E. Brothers, K. N. Kudin, V. N. Staroverov, R. Kobayashi, J. Normand, K. Raghavachari, A. Rendell, J. C. Burant, S. S. Iyengar, J. Tomasi, M. Cossi, N. Rega, J. M. Millam, M. Klene, J. E. Knox, J. B. Cross, V. Bakken, C. Adamo, J. Jaramillo, R. Gomperts, R. E. Stratmann, O. Yazyev, A. J. Austin, R. Cammi, C. Pomelli, J. W. Ochterski, R. L. Martin, K. Morokuma, V. G. Zakrzewski, G. A. Voth, P. Salvador, J. J. Dannenberg, S. Dapprich, A. D. Daniels, Ö. Farkas, J. B. Foresman, J. V. Ortiz, J. Cioslowski, and D. J. Fox, Gaussian, Inc., Wallingford CT, 2009.

4) D. Rehm and A. Weller, *Isr. J. Chem.* 1970, **8**, 259–271.

5) The free energy change for charge separation (ΔG_{CS}) from the singlet excited state ^{1}ZnP* within the donor-acceptor system is calculated using spectroscopic, computational and electrochemical data following equations 1-3.

$$-\Delta G_{CR} = E_{ox} - E_{red} + \Delta G_S \qquad (1)$$

$$-\Delta G_{CS} = \Delta E_{00} - (-\Delta G_{CR}) \qquad (2)$$

where ΔE_{00} and ΔG_{CS} correspond to the energy of excited singlet state (2.04 eV) and electrostatic energy, respectively. The E_{ox} and E_{red} represent the oxidation potential of the electron donor, ZnP and the reduction potential of the electron acceptor CuC, respectively. The term ΔG_s refers to the static Coulombic energy, calculated by using the “dielectric continuum model” according to equation (3):

$$\Delta G_S = \frac{e^2}{4\pi\varepsilon_0}\left[\left(\frac{1}{2R_+} + \frac{1}{2R_-}\right)\Delta\left(\frac{1}{\varepsilon_R}\right) - \frac{1}{R_{CC}\varepsilon_R}\right] \qquad (3)$$

The symbols ε_0, and ε_R represent vacuum permittivity and dielectric constant of the solvent used for photochemical and electrochemical studies, respectively. R_{CC} is the center-to-center distance between donor and acceptor entities. The symbols R_+ and R_- refer to radii of the cation and anion species, respectively. The calculated ΔG_S was found to be 0.08 eV for the present donor-acceptor system.

6) A. Poater, B. Cosenza, A. Correa, S. Giudice, F. Ragone, V. Scarano and L. Cavallo, *Eur. J. Inorg. Chem.* 2009, 1759

7) P. N. Taylor and H. L. Anderson, *J. Am. Chem. Soc.*, **1999**, 121, 11538-11545.

8) J. W Akitt and B. E. Mann, B. E. *NMR and Chemistry: An Introduction to Modern NMR Spectroscopy, 4th ed.*, pp. 69–73 (Stanley Thornes Publishers Ltd., Cheltenham, 2000).
